# *Centaurea pungens* Pomel as a potential nutraceutical source: Polyphenol-rich profile, antioxidant capacity, and inhibition of digestive α-amylase

**DOI:** 10.3389/fnut.2026.1866954

**Published:** 2026-08-28

**Authors:** Randa Mlik, Mohamed Amine Fares, Nour Elhouda Mekhadmi, Asma Abid, Assia Bentahar, Walid Boussebaa, Slimane Ahmed Benmchih Ayada, Mohamed Kharsi, Makhlouf Sekour, Samia Hamouda, Wafa Ali Eltayb, Han Yu, Mohnad Abdalla, Ali Alsalme, Mikhael Bechelany, Ahmed Barhoum

**Affiliations:** 1 Higher School of Saharan Agriculture, El Oued, Algeria; 2 Saharan Area Laboratory for Modernization and Development of Agriculture (SALAMA Lab), Higher School of Saharan Agriculture, El Oued, Algeria; 3 Laboratory of the Development and Technology of Saharan Resources (VTRS), University of El Oued, El Oued, Algeria; 4 Department of Biology, Faculty of Natural and Life Sciences, University of El Oued, El Oued, Algeria; 5 Laboratory of Valorization and Promotion of Saharan Resources (VPRS), Faculty of Mathematics and Matter Sciences, University of Ouargla, Ouargla, Algeria; 6 Laboratory of Phytotherapy Applied to Chronic Diseases, SNV Faculty, University of Setif, Setif, Algeria; 7 Scientific and Technical Research Center in Physico-Chemical Analysis (CRAPC), Headquarters Ex-Pasna Industrial Zone, Bou-Ismail, Tipaza, Algeria; 8 National Institute of Agronomic Research of Algeria, Station of Adrar, Adrar, Algeria; 9 Department of Agronomic Sciences, Faculty of Life and Natural Sciences, Kasdi-Merbah University, Ouargla, Algeria; 10 Anesthesiology and ICU Department, Faculty of Medicine for Girls, Al-Azhar University, Cairo, Egypt; 11 Biotechnology Department, Faculty of Science and Technology, Shendi University, Shendi, Sudan; 12 Pediatric Research Institute, Children's Hospital Affiliated to Shandong University, Jinan, Shandong, China; 13 Research Institute, Children's Hospital Affiliated to Shandong University, Jinan, Shandong, China; 14 Department of Chemistry, College of Science, King Saud University, Riyadh, Saudi Arabia; 15 Institut Européen des Membranes, IEM, UMR 5635, University Montpellier, ENSCM, CNRS, Montpellier, France; 16 Department of Mathematics and Natural Sciences, Gulf University for Science and Technology (GUST), Hawally, Kuwait; 17 School of Chemical and BioPharmaceutical Sciences, Technological University Dublin, Dublin, Ireland

**Keywords:** *Centaurea pungens*, functional food, metabolic health, nutraceutical, polyphenols, α-amylase inhibition

## Abstract

**Introduction:**

Antioxidant-rich plant-derived compounds are increasingly investigated for their role in preventing oxidative stress and managing metabolic disorders. *Centaurea pungens* Pomel, an underexplored Saharan species, represents a potential source of dietary bioactive compounds with functional properties.

**Methods:**

This study aimed to evaluate the phytochemical composition and nutraceutical potential of *C. pungens* through integrated chemical, biological, and in silico approaches.

**Results:**

The ethanolic extract was characterized using UPLC-ESI-MS/MS, revealing a diverse phytochemical profile comprising flavonoids and phenolic acids, with major constituents including curcumin, beta-carotene, and myricetin derivatives. The extract exhibited notable antioxidant activity, with 2,2-Diphenyl-1-picrylhydrazyl (DPPH) radical scavenging (IC50 = 0.0706 ± 0.006 mg/mL) and hydrogen peroxide scavenging (IC50 = 0.1789 ± 0.083 mg/mL), alongside moderate metal-chelating capacity. The extract exhibited significant in vitro alpha-amylase inhibitory activity. Molecular docking and 300 ns molecular dynamics simulations further suggested that myricetin 3-arabinoside may contribute to the observed inhibitory effect through stable interactions with the catalytic pocket of human pancreatic alpha-amylase. In addition, the extract showed antimicrobial activity, indicating its potential for applications related to food safety and preservation.

**Discussion:**

However, the predominant nutritional relevance lies in its high polyphenol content and associated antioxidant and enzyme-inhibitory properties. Overall, these findings suggested that *C. pungens* represents a promising source of bioactive phytochemicals deserving further nutritional investigation. Further quantitative phytochemical analysis, together with bioavailability, safety, and in vivo efficacy studies, is warranted before practical dietary applications can be recommended.

## 1 Introduction

Dietary patterns rich in plant-derived bioactive compounds are increasingly recognized for their role in reducing the risk of chronic diseases, particularly those associated with oxidative stress and metabolic dysfunction (1, 2). Polyphenols, flavonoids, and carotenoids contribute to these protective effects through their antioxidant properties and their capacity to modulate key metabolic enzymes involved in carbohydrate digestion and glucose homeostasis (3). In this context, the identification and characterization of novel plant-based sources of such compounds have become central to the development of functional foods and nutraceuticals targeting metabolic health. Arid and semi-arid ecosystems constitute an underexplored reservoir of nutritionally relevant phytochemicals. Plants adapted to extreme environmental conditions often accumulate elevated levels of secondary metabolites with potent biological activities, including antioxidant and enzyme inhibitory properties (4, 5). Within this framework, species within the genus *Centaurea*, family Asteraceae, are recognized for their richness in phenolic compounds and flavonoids, which are associated with traditional uses in the management of metabolic and inflammatory disorders (6, 7). However, despite growing interest in this genus, several endemic species remain insufficiently characterized from a nutritional and functional standpoint.

The genus *Centaurea* L. is among the most taxonomically and ethnobotanically significant groups in Asteraceae, comprising over 500 species distributed across Europe, the Mediterranean, Western Asia, and North Africa (8). In Algeria, 45 species are recorded, seven of which are endemic to the Saharan desert, reflecting remarkable adaptation to arid habitats (9). Traditionally, *Centaurea* species have been used to treat inflammation, diabetes, gastrointestinal disorders, cardiovascular diseases, and infections (10). Phytochemically, they are rich in flavonoids, sesquiterpene lactones, and phenolic acids, which also serve as chemotaxonomic markers (11). For instance, *C. parviflora* extracts demonstrated antioxidant and antimicrobial activity against pathogens such as *S. aureus, E. coli, P. aeruginosa*, and *K. pneumoniae*, linked to their high phenolic and flavonoid content (12). These studies indicate that *Centaurea* species are a valuable source of compounds capable of modulating oxidative stress, inhibiting microbial growth, and exerting multiple pharmacological effects, making them prime candidates for systematic biological and chemical investigation.

Sesquiterpene lactones (SLs) are the most chemically distinctive and biologically relevant class within *Centaurea*, characterized by an α-methylene-γ-lactone moiety that reacts with nucleophilic cellular targets, including thiol-containing enzymes, producing antimicrobial, anti-inflammatory, antitumor, antidiabetic, and antifungal effects (13, 14). More than 8,000 SLs have been described, with Asteraceae as their main botanical source (13). For example, LC-MS profiling of *C. parviflora* identified rutin, quercetin-3-O-glucoside, chlorogenic acid derivatives, and quinic acid, which efficiently scavenge reactive oxygen species, modulate enzymes, and inhibit microbial growth (10). Total phenolic content strongly correlates with antioxidant capacity in *Centaurea* species, as demonstrated using 2,2-Diphenyl-1-picrylhydrazyl (DPPH), 2,2′-Azino-bis(3-ethylbenzothiazoline-6-sulfonic acid) (ABTS), Ferric Reducing Antioxidant Power (FRAP), and Cupric Reducing Antioxidant Capacity (CUPRAC) assays. This combination of bioactive secondary metabolites explains the broad pharmacological activities observed and highlights the potential of unexplored species like *C. pungens* to yield novel therapeutics and natural antimicrobial agents. In addition to their pharmacological importance, sesquiterpene lactones have gained increasing attention as bioactive phytochemicals with potential nutritional relevance. Their antioxidant, anti-inflammatory, antimicrobial, and enzyme-modulating properties suggest that they may contribute to the health benefits associated with plant-based diets and medicinal plants. Consequently, these compounds are increasingly being explored as potential nutraceutical ingredients and functional food components for supporting metabolic health and preventing oxidative stress-related disorders.

*C. pungens* Pomel, an endemic herb of the Algerian Sahara, remains poorly studied despite its traditional use as an antidiabetic, antimicrobial, and condiment plant (9). Previous studies isolated novel sesquiterpene lactone conjugates, flavonoids, and quinic acid derivatives, showing initial antimicrobial activity, but comprehensive profiling of their antioxidant, antibacterial, antifungal, and enzyme inhibitory properties is lacking. The present study addresses this gap by integrating UPLC-ESI-MS/MS phytochemical analysis, computational pharmacology (molecular docking and dynamics), and experimental validation, including antioxidant assays (DPPH, H_2_O_2_, iron chelation, FTC, TBA) and antimicrobial testing against ESKAPE pathogens (*Enterococcus faecium, Staphylococcus aureus, Klebsiella pneumoniae, Acinetobacter baumannii, Pseudomonas aeruginosa* and *Enterobacter* spp.), and phytopathogens. This approach allows the identification of key bioactive compounds, such as Myricetin 3-arabinoside, and links their chemical properties to functional activities. To our knowledge, this is the first comprehensive investigation integrating phytochemical profiling, antioxidant evaluation, antimicrobial assays, experimental α-amylase inhibition, and molecular simulations to characterize the nutraceutical potential of *C. pungens*. The study's findings are significant for pharmaceutical and agricultural applications, supporting the development of natural antioxidants, antimicrobial therapeutics, and biopesticides derived from an arid-zone medicinal plant adapted to extreme environmental stress.

## 2 Materials and methods

### 2.1 Plant material and extract preparation

Aerial parts of *C. pungens* Pomel were collected from Adrar Province, southwestern Algeria, during the 2024–2025 growing season. Botanical identification was confirmed by Professor EDDOUD Amar from the Department of Agronomic Sciences, Faculty of Natural and Life Sciences, University of Ouargla, Algeria. A voucher specimen (HSSA/PP/01/26) was deposited at the Herbarium of the Higher School of Saharan Agriculture, El Oued, Algeria.

The plant material was oven-dried at 45 °C for 24 h until a constant weight was achieved. The dried material was ground to a fine powder using a mechanical grinder. For extraction, 30 g of powdered material was macerated in 100 mL of 70% ethanol. The mixture was continuously agitated at 120 rpm in complete darkness at 25 ± 2 °C for 5 days. Following maceration, the extract was filtered through Whatman No. 1 filter paper, and the filtrate was concentrated under reduced pressure at 45 °C using a rotary evaporator (R-300, Büchi, Switzerland) to obtain a dry ethanolic extract. The extract was stored at 4 °C in amber vials until further analysis. The extraction yield (%) was calculated as:


$$\begin{array}{l} \text{Yield }(\%)\text{=}\frac{\text{Weight of dry extract }(\text{g})}{\text{Weight of dry plant material }(\text{g})}\times \text{100} \end{array}$$

All extractions were performed in triplicate to ensure reproducibility.

### 2.2 Determination of total phenolic content (TPC) of the extract

The total phenolic content of the *C. pungens* ethanolic extract was determined using the Folin-Ciocalteu method with minor modifications from Li et al. (15). Briefly, 0.1 mL of extract solution (1 mg/mL in ethanol) was mixed with 0.5 mL of 10-fold diluted Folin-Ciocalteu reagent. The mixture was incubated at 25 ± 2 °C for 4 min in the dark. Subsequently, 0.4 mL of 7.5% Na_2_CO3 solution was added, and the reaction was further incubated in darkness for 90 min. Absorbance was measured at 760 nm using a UV-Vis spectrophotometer. Gallic acid was used as a calibration standard (0–200 μg/mL), and TPC was expressed as mg gallic acid equivalents per gram of dry extract (mg GAE/g DW). All measurements were performed in triplicate.

### 2.3 Determination of total flavonoid content (TFC) of the extract

Total flavonoid content was quantified following the method of Bahorun et al. (16). In brief, 1 mL of extract solution (1 mg/mL in methanol) was mixed with 1 mL of 2% AlCl3 solution. The reaction was allowed to proceed at 25 ± 2 °C for 10 min in the dark. Absorbance was measured at 430 nm against a reagent blank lacking extract. Quercetin (0–100 μg/mL) was used as a standard, and TFC was expressed as mg quercetin equivalents per gram of dry extract (mg QE/g DW). Measurements were performed in triplicate.

### 2.4 UPLC-ESI-MS/MS analysis of the extract

The chemical profile of *C. pungens* ethanolic extract was analyzed using a Shimadzu LC-MS/MS 8040 system equipped with an electrospray ionization (ESI) source. Chromatographic separation was performed on a Restek Ultra C18 column (3 μm, 150 × 4.6 mm). The mobile phase consisted of solvent A (water with 0.1% formic acid) and solvent B (methanol), delivered at a flow rate of 0.2 mL/min. The chromatographic separation was performed using a gradient elution program in which solvent A was maintained at 98% from 0 to 0.2 min, then linearly decreased to 25% between 0.2 and 2.5 min, followed by a further linear reduction to 0% from 2.5 to 4 min. The composition was held at 0% A from 4 to 7 min, then rapidly returned to 98% A between 7 and 7.1 min and finally maintained at 98% A until 12 min to allow for column re-equilibration. The ESI-MS/MS analysis was conducted under the following conditions: the collision-induced dissociation (CID) gas was set to 230 kPa, the conversion dynode voltage was −6.0 kV°C, and the desolvation line (DL) temperature was maintained at 250 °C. Nebulizing gas was delivered at 3.0 L/min, the heat block temperature was set to 400 °C, and the drying gas flow was 10 L/min, ensuring optimal ionization and reproducible detection of analytes. Compound identification was performed by comparing the retention times and multiple reaction monitoring (MRM) transitions of the detected analytes with those of authentic reference standards analyzed under identical chromatographic and mass spectrometric conditions. The identity of each metabolite was confirmed only when both the retention time and the corresponding MRM transitions matched those of its authentic standard.

### 2.5 Molecular docking and *in silico* methodology

The 3D crystal structure of Human Pancreatic α-Amylase (HPA; PDB ID: 3BAJ) was retrieved from the Protein Data Bank. Protein preparation followed standard protocols: water molecules and heteroatoms were removed, missing residues were modeled, and hydrogen atoms were added. Protonation states of ionizable residues were optimized for physiological pH (7.4) to ensure accurate modeling of electrostatic interactions within the active site. Candidate ligands were obtained from the PubChem database and converted to three-dimensional structures. Energy minimization was performed using the MMFF94 force field, and rotatable bonds were optimized to allow conformational flexibility during docking. A co-crystallized ligand was used as a reference to validate the docking protocol.

Molecular docking was conducted using AutoDock Vina to predict binding orientations and interactions within the HPA catalytic site. The grid box was centred on the active site cleft, encompassing key residues Asp197, Glu233, Asp300, Arg195, His299, and His305. Binding energies, hydrogen bonds, and hydrophobic contacts were analyzed, and docking poses were visualized in both two- and three-dimensional diagrams to assess geometric and chemical complementarity between ligands and the binding pocket.

To further evaluate the dynamic stability of protein-ligand complexes, 300 ns molecular dynamics (MD) simulations were carried out using GROMACS with the AMBER99SB-ILDN force field. Complexes were solvated in a TIP3P water box with a 10 Å buffer, neutralized with counter ions, and subjected to energy minimization using the steepest descent algorithm. Equilibration was performed sequentially under NVT and NPT ensembles for 1 ns each at 310 K, followed by production MD runs of 300 ns. Trajectories were analyzed to calculate root mean square deviation (RMSD) for overall structural stability, root mean square fluctuation (RMSF) for residue flexibility, and radius of gyration (Rg) to evaluate structural compactness.

Protein-ligand interactions were quantified over the MD trajectories using interaction fingerprints, including hydrogen bonds, hydrophobic contacts, ionic interactions, and water bridges. Interaction fractions were calculated to determine the persistence of contacts with key catalytic residues throughout the simulation. Binding free energies were estimated using the MM-GBSA approach, with contributions from van der Waals, electrostatic, hydrogen bonding, lipophilic, and solvation energies summed to obtain the total binding free energy (ΔGbind).

### 2.6 *In vitro* evaluation of α-amylase inhibitory activity using the starch–iodine microplate assay

The starch–iodine assay was selected because it provides a rapid and reproducible colorimetric assessment of α-amylase activity and has been widely applied for screening natural products with potential antidiabetic properties. Although less specific than chromatographic approaches, it is appropriate for preliminary evaluation of crude plant extracts.

The α-amylase inhibitory activity of the ethanolic extract of *C. pungens* was evaluated using a modified starch–iodine colorimetric assay in a 96-well microplate. The assay was based on the inhibition of starch hydrolysis by α-amylase, where the remaining starch forms a blue complex with iodine. The absorbance was measured at 620–630 nm as an indirect indicator of enzyme inhibition. A 20 mM sodium phosphate buffer (pH 6.9) containing 6 mM NaCl was used for the assay. Porcine pancreatic α-amylase solution and freshly prepared 1% (w/v) soluble starch were used as the enzyme and substrate, respectively. The iodine reagent consisted of 5 mM I_2_ and 5 mM KI. The ethanolic extract was dissolved in 1% DMSO and diluted with phosphate buffer to obtain final concentrations of 80, 40, 20, 10, 5, 2.5, and 1.25 mg/mL. For each well, 120 μL of phosphate buffer, 20 μL of extract solution, and 20 μL of α-amylase solution were mixed and pre-incubated at 37 °C for 10 min. Subsequently, 20 μL of 1% soluble starch was added, and the reaction was incubated for 15 min at 37 °C. The reaction was stopped by adding 20 μL of 1 M HCl, followed by 100 μL of iodine reagent. The absorbance was then measured at 620–630 nm using a microplate reader.

Acarbose served as the positive control, whereas the reaction mixture without extract was used as the negative control. Extract blanks (without enzyme) and a vehicle control (1% DMSO) were included to eliminate background absorbance and confirm the absence of solvent interference. The percentage inhibition of α-amylase activity was calculated using the following equations:


$$\begin{array}{lll} \text{Δ}{\text{A}}_{\text{control}} & = & A_{\text{test}}-A_{\text{blank}}\\ \Delta A_{\text{sample}} & = & A_{\text{sample}}-A_{\text{sampleblank}}\\ \%\text{ Inhibition} & = & \left[\left(\Delta A_{\text{control}}-\Delta A_{\text{sample}}\right)/\Delta A_{\text{control}}\right]\;\times \;100 \end{array}$$

The IC50 value was determined by nonlinear regression analysis of the inhibition percentages against the logarithm of extract concentrations. All experiments were performed in triplicate (*n* = 3), and the results were expressed as mean ± standard deviation (SD), providing a preliminary evaluation of the antidiabetic potential of the extract through α-amylase inhibition (17).

### 2.7 Antioxidant activities

The radical scavenging potential of *C. pungens* ethanolic extract was evaluated using multiple complementary assays, including DPPH, hydrogen peroxide, iron chelation, ferric thiocyanate (FTC), and thiobarbituric acid (TBA) methods. For DPPH radical scavenging, 50 μL aliquots of extract at varying concentrations were mixed with 5 mL of 0.004% DPPH solution in methanol and incubated in the dark at 25 ± 2 °C for 30 min. Absorbance was measured at 517 nm, with butylated hydroxytoluene (BHT) serving as a positive control. Antioxidant activity was expressed as percentage inhibition (I%) according to the formula:


$$\begin{array}{l} I\%=100\times \frac{A_{\text{control}}-A_{\text{sample}}}{A_{\text{control}}} \end{array}$$

Hydrogen peroxide scavenging activity was assessed by combining 380 μL of extract at different concentrations with 62.5 μL of ammonium solution, followed by the addition of 17.5 μL of H_2_O_2_. The mixture was incubated at 25 ± 2 °C for 5 min, then 380 μL of ferroin reagent was added, and the reaction continued for an additional 10 min. Absorbance was recorded at 230 nm, and activity was expressed relative to vitamin C prepared under identical conditions.

Iron-chelating capacity was determined using the Fe^2+^-ferrozine assay adapted from Decker and Welch (18). In brief, 40 μL of methanol, 40 μL of extract solution, 40 μL of 0.2 mM Fe^2+^, and 80 μL of ferrozine were sequentially mixed. The reaction was incubated for 10 min at room temperature, and absorbance was measured at 593 nm. EDTA was used as a positive control, and chelation efficiency (%) was calculated as:


$$\begin{array}{l} \text{Iron chelation }\left(\%\right)=\frac{A_{\text{control}}-A_{\text{extract}}}{A_{\text{control}}}\times 100 \end{array}$$

The antioxidant effect on lipid peroxidation was evaluated using both the FTC and TBA methods. For the FTC assay, a linoleic acid emulsion was prepared by emulsifying 0.2804 g linoleic acid and 0.2804 g Tween 20 in 50 mL of 0.2 M phosphate buffer (pH 7.0). Reaction mixtures containing 0.5 mL of extract, 2.5 mL of linoleic acid emulsion, and 2 mL of phosphate buffer were incubated at 37 °C for 5 days. Daily, 0.1 mL aliquots were mixed with 4.7 mL of 75% ethanol, 0.1 mL of 30% ammonium thiocyanate, and 0.1 mL of 0.02 M FeCl_2_ in 3.5% HCl. After 3 min, absorbance was measured at 500 nm, with BHT and vitamin C as reference standards.

On the final day, thiobarbituric acid reactive substances (TBARS) were quantified to assess secondary lipid peroxidation products. One milliliter of the reaction mixture was combined with 2 mL of 20% trichloroacetic acid and 2 mL of TBA solution, then heated in a boiling water bath (100 °C) for 10 min. After centrifugation at 3,000 rpm for 20 min, absorbance of the supernatant was measured at 532 nm. Antioxidant activity was expressed as percentage inhibition relative to the final FTC readings, using BHT and vitamin C as positive controls. All assays were performed in triplicate to ensure reproducibility, and results are reported as mean ± standard deviation.

### 2.8 Antimicrobial activities

The antibacterial activity of *C. pungens* ethanolic extract was assessed against bacterial strains obtained from the Pasteur Institute of Algeria, including four Gram-positive species (*Staphylococcus aureus* ATCC 2592, *S. aureus* ATCC 29213, *Enterococcus faecalis* ATCC 49532, and *Bacillus subtilis* ATCC 6633) and three Gram-negative species (*Pseudomonas aeruginosa* ATCC 27853, *Escherichia* coli ATCC 25922, and *Klebsiella pneumoniae* ATCC 70603). Müller-Hinton agar plates were prepared, allowed to solidify, and surface-dried before inoculation. Stock extract solutions (100 mg/mL) were prepared in 10% DMSO, and sterile 5 mm discs were loaded with 50, 75, or 100 μL of extract dilutions. DMSO alone served as a negative control, while penicillin (10 μg), rifampicin (5 μg), and amoxicillin (25 μg) were used as positive controls. Plates were incubated at 37 °C for 18–24 h, and inhibition zone diameters (IZ) were measured. Antibacterial sensitivity was classified according to IZ diameter as not sensitive (< 8 mm), sensitive (9–14 mm), very sensitive (15–19 mm), or extremely sensitive (>20 mm). All assays were performed in triplicate to ensure reproducibility.

Antifungal activity was evaluated by determining the minimum inhibitory concentration (MIC) and minimum fungicidal concentration (MFC) using a modified agar diffusion protocol (19). A stock solution of extract (100 mg/mL in 10% DMSO) was serially diluted to six concentrations (100, 75, 50, 25, 12.5, and 6.25 mg/mL). Aliquots (50 μL) were evenly applied to solidified potato dextrose agar (PDA) plates and air-dried for 20–30 min. Mycelial plugs (5 mm diameter) from 7-day-old cultures of *Fusarium oxysporum, Alternaria alternata*, and *A. solani* were centrally inoculated under aseptic conditions. Plates (*n* = 3 per concentration) and PDA-only controls were incubated at 27 °C until complete colonization of the control plates. MIC was defined as the lowest concentration preventing visible mycelial growth. MFC was confirmed by subculturing non-growing mycelia onto antifungal-free PDA, with the absence of regrowth after 48 h indicating fungicidal activity. Mycelial inhibition (%) was calculated using:


$$\begin{array}{l} \text{Inhibition }(\%)\text{=}\frac{\text{Control diameter-Treatment diameter}}{\text{Control diameter}}\times 100 \end{array}$$

Mycelial growth speed (GS) was determined according to Bakan et al. (20) as the cumulative daily growth:


$$\begin{array}{l} GS=\frac{D_{1}}{Te_{1}}+\frac{D_{2}-D_{1}}{Te_{2}}+\cdots +\frac{D_{n}-D_{n-1}}{Te_{n}} \end{array}$$

where D represents daily mycelial growth diameter and Te corresponds to the incubation time.

### 2.9 Statistical analysis

All experiments were conducted in triplicate in a randomized order, and results are expressed as mean ± standard deviation. Statistical analyses were performed using R software (version 4.2.3). Normality of dependent variables was evaluated using the Shapiro-Wilk test for datasets with *n* < 50. One-way ANOVA was applied for normally distributed data, whereas non-parametric data were analyzed using the Kruskal-Wallis test. Pearson or Spearman correlation analyses were performed to assess relationships between *C. pungens* phytochemical parameters and measured biological activities. Statistical significance was considered at α = 0.05 and 0.01.

## 3 Results and discussion

### 3.1 Total phenol (TPC) and flavonoid (TFC) contents

Phytochemical analysis of *C. pungens* ethanolic extract revealed a high total phenolic content (TPC) of 74.91 ± 0.46 mg GAE/g DW. The total flavonoid content (TFC) was comparatively lower at 5.63 ± 0.08 mg QE/g DW. The significantly higher TPC compared to TFC (*p* < 0.01, Student's *t*-test) suggests that phenolic compounds are the predominant class of secondary metabolites in this species. This high phenolic content may underlie the extract's strong antioxidant and antimicrobial potential.

The predominance of total phenolics over flavonoids indicates that non-flavonoid phenolic constituents, including phenolic acids and related derivatives, probably represent a substantial fraction of the extract. Such compounds are well known to contribute to antioxidant activity through electron donation, free radical scavenging, and transition-metal chelation, suggesting that they may largely account for the biological properties observed in the present study.

This pattern is consistent with findings from other *Centaurea* species. *C. parviflora* extracts showed comparable trends with TPC values of 0.17527 ± 0.00279 mg GAE/mg but lower flavonoid content of 0.05989 ± 0.00091 mg QE/mg (10), indicating that phenolic acids and related polyphenolic compounds are characteristic bioactive constituents across the genus. Similarly, *Centaurea lycaonica* demonstrated that methanol extracts possessed high total phenolic and flavonoid contents, with phenolic compounds and flavonoids identified as the main represented classes, supporting the predominance of phenolic acids over other phenolic subclasses (21). Additionally, *C. cyanus* and *C. calcitrapa* exhibited TPC values of 40–80 mg GAE/g DW, confirming that *Centaurea* species are rich in phenolics with bioactive potential (22–24). In contrast, the TFC in *C. pungens* was lower than in species such as *C. solstitialis* (12–18 mg QE/g DW) (25), reflecting interspecies variability and potential effects of geographic origin, harvesting time, and extraction solvent. The high TPC in *C. pungens* is particularly noteworthy, as phenolics are known to scavenge free radicals, chelate metals, and inhibit microbial growth (10).

### 3.2 UPLC-ESI-MS-MS analysis

**Figure 1:**
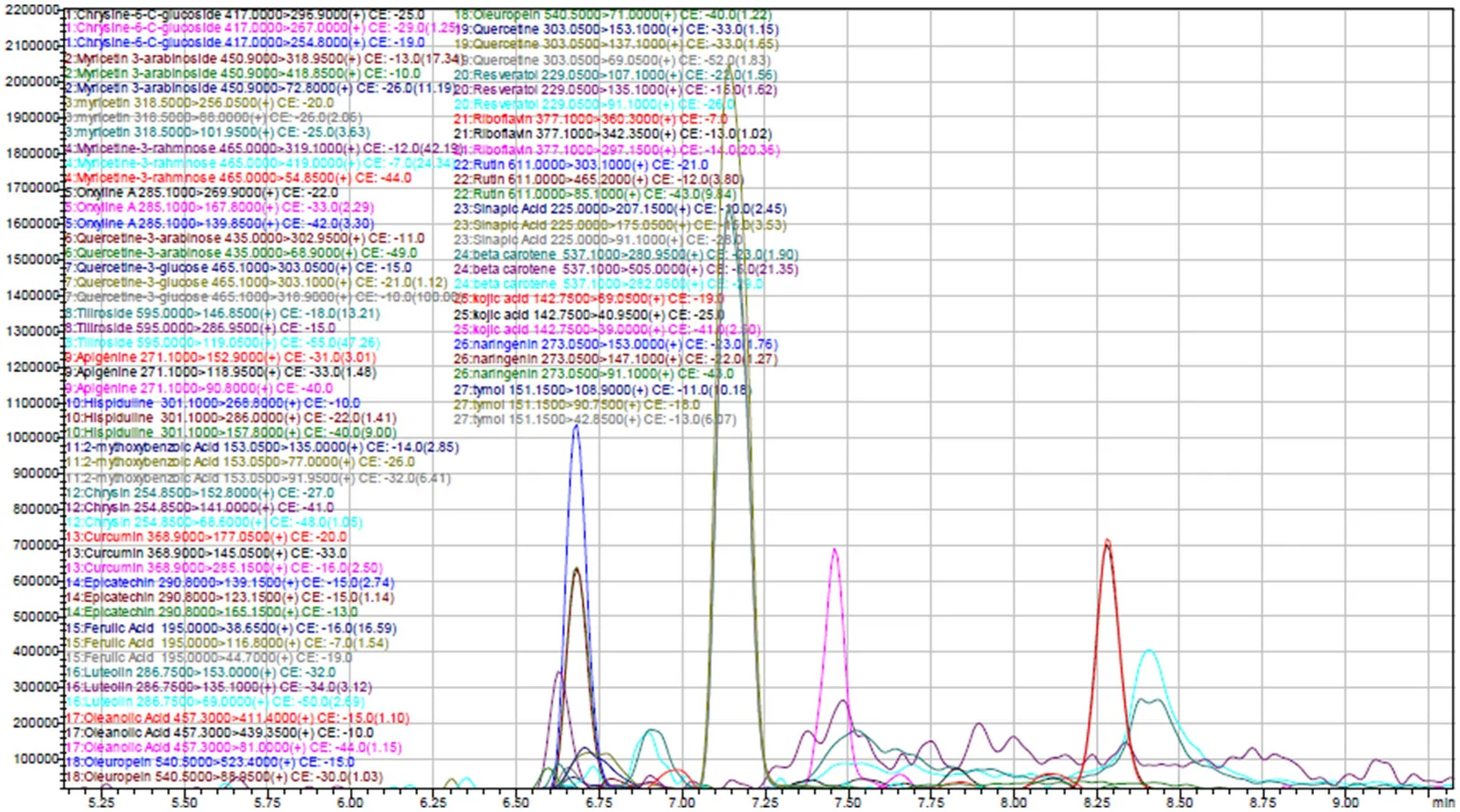
UPLC-ESI–MS/MS chromatogram of *Centaurea pungens* extract.

**Figure 2:**
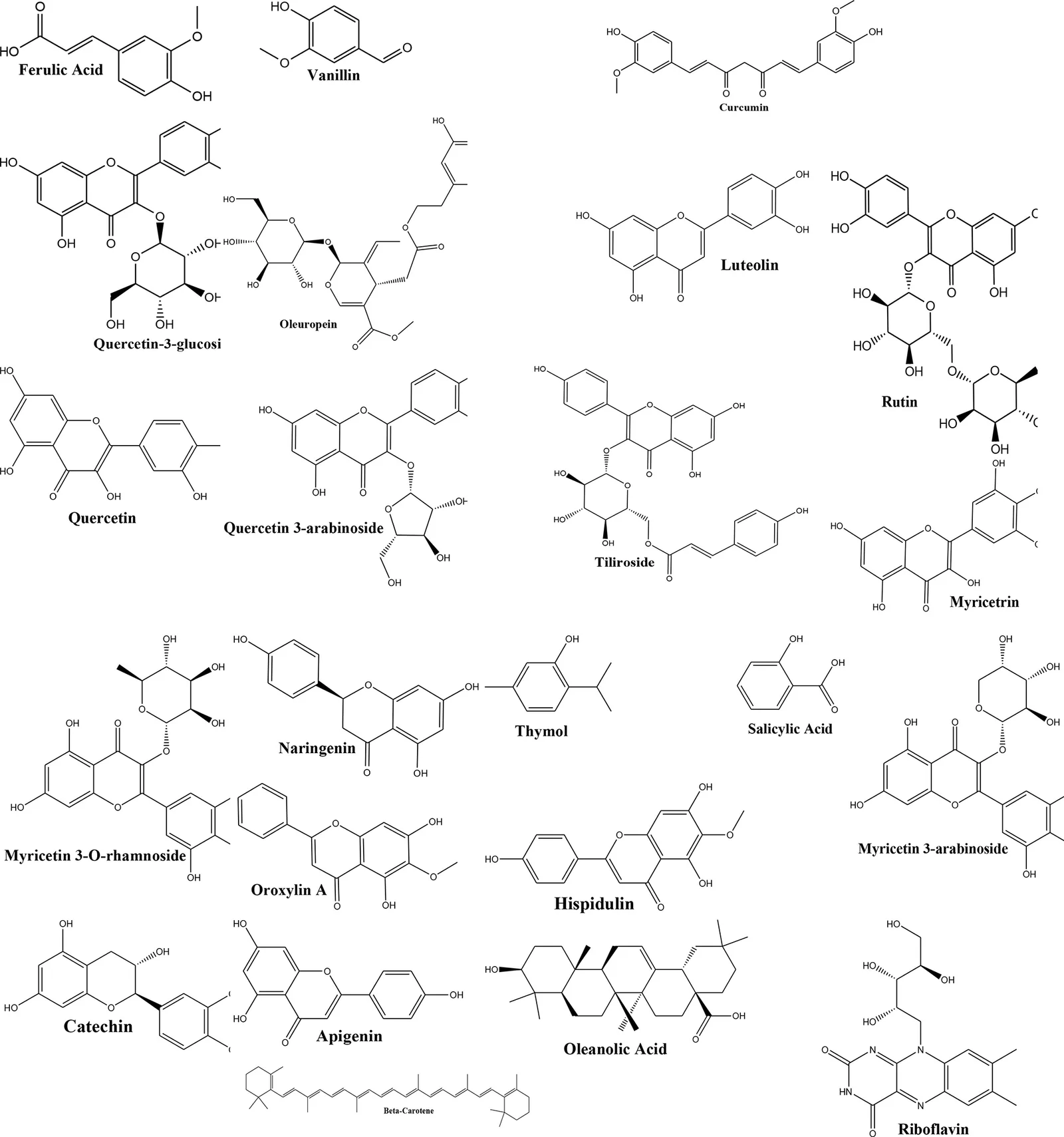
Chemical structures of the compounds identified in *Centaurea pungens* extract.

UPLC-ESI-MS/MS profiling revealed a diverse phytochemical profile of *C. pungens* extract (Table 1, Figures 1, 2). The chromatogram showed well-resolved peaks, indicating metabolites with varying polarity and structural complexity. Flavonoids and their glycosides were the dominant class, including quercetin, luteolin, apigenin, naringenin, rutin, quercetin-3-glucoside, and multiple myricetin derivatives. Phenolic acids such as ferulic acid, vanillin, and salicylic acid were also present. Other bioactive compounds included curcumin, oleuropein, catechin, thymol, riboflavin, oleanolic acid, and β-carotene. Peak area counts indicated relative abundance, indicating that curcumin (4.57 × 106), β-carotene (2.65 × 106), and myricetin-3-arabinoside (2.37 × 106) were the most abundant metabolites. The high concentrations of these compounds were statistically significant (*p* < 0.01), highlighting their potential contribution to antioxidant and antimicrobial activity. Glycosylated flavonoids, such as quercetin-3-glucoside and myricetin derivatives, were also present at notable levels (*p* < 0.05), suggesting synergistic effects with other phenolics.

**Table 1:** UPLC-ESI–MS/MS identification of phytochemical constituents in *C. pungens* extract.

Compound	Molecular formula	Rt (min)	ESI mode	MRM transition (m/z)
Ferulic acid	C_10_H_10_O_4_	5.673	+	195.0000 → 44.7000
Vanillin	C_8_H_8_O_3_	6.192	–	153.0500 → 92.8500
Curcumin	C_21_H_20_O_6_	6.465	+	368.9000 → 177.0500
Oleuropein	C_25_H_32_O_13_	6.648	+	540.5000 → 523.4000
Rutin	C_27_H_30_O_16_	6.681	+	611.0000 → 303.1000
Quercetin	C_15_H_10_O_7_	6.686	+	303.0500 → 153.1000
Quercetin-3-glucoside	C_21_H_19_O_12_	6.707	+	465.1000 → 303.0500
Quercetin-3-arabinoside	C_20_H_18_O_11_	6.788	+	435.0000 → 302.9500
Thymol	C_10_H_14_O	6.877	+	151.1500 → 90.7500
Tiliroside	C_30_H_26_O_13_	6.907	+	595.0000 → 146.8500
Myricetin 3-*O*-rhamnoside	C_21_H_20_O_12_	6.986	+	465.0000 → 54.8500
Luteolin	C_15_H_10_O_6_	7.044	+	286.7500 → 153.0000
Naringenin	C_15_H_12_O_5_	7.057	+	273.0500 → 153.0000
Salicylic acid	C_7_H_6_O_3_	7.093	–	137.2000 → 92.9500
Myricetin	C_15_H_10_O_8_	7.141	+	318.5000 → 256.0500
Hispidulin	C_16_H_12_O_6_	7.259	+	301.1000 → 268.8000
Oroxylin A	C_16_H_12_O_5_	7.404	+	285.1000 → 269.9000
Myricetin-3-arabinoside	C_20_H_18_O_12_	7.742	+	450.9000 → 418.8500
Oleanolic acid	C_30_H_48_O_3_	8.106	+	457.3000 → 411.4000
Apigenin	C_15_H_10_O_5_	8.140	+	271.1000 → 90.8000
Catechin	C_15_H_14_O_6_	8.166	+	291.1000 → 161.1000
Riboflavin	C_17_H_20_N_4_O_6_	8.283	+	377.1000 → 360.3000
β-Carotene	C_40_H_56_	8.384	+	537.1000 → 280.9500

Rt, retention time; ESI, electrospray ionization; MRM, multiple reaction monitoring; m/z, mass-to-charge ratio.

Beyond identifying individual metabolites, the phytochemical profile indicates that *C. pungens* possesses remarkable chemical diversity, including flavonoids, phenolic acids, carotenoids, and other secondary metabolites with complementary biological functions. Such chemical diversity may contribute to the multifunctional properties of the extract through additive or complementary mechanisms rather than through the action of a single constituent. These findings align with reports on related *Centaurea* species, where flavonoids and phenolic acids were associated with strong radical scavenging and antimicrobial effects (23). Curcumin and myricetin derivatives are well-documented for antioxidant, enzyme inhibitory, and antimicrobial properties (26–28). The combination of high-abundance bioactive compounds and structural diversity provides a chemical rationale for the potent biological activities observed in *C. pungens* extract.

It is important to acknowledge that the UPLC-ESI-MS/MS analysis employed in this study provides qualitative and semi-quantitative information based on peak area counts, which reflect relative abundance rather than absolute concentrations. While this approach successfully identified the phytochemical diversity of the extract and highlighted the most prominent metabolites, it does not establish the precise quantitative contribution of individual compounds to the total extract mass. Consequently, the concentrations of specific bioactives such as myricetin 3-arabinoside, curcumin, or β-carotene within the extract remain undetermined. This represents a methodological limitation that should be considered when interpreting the biological activities observed in this study.

### 3.3 *In silico* analysis

#### 3.3.1 Initial molecular docking and binding pocket topography

**Figure 3:**
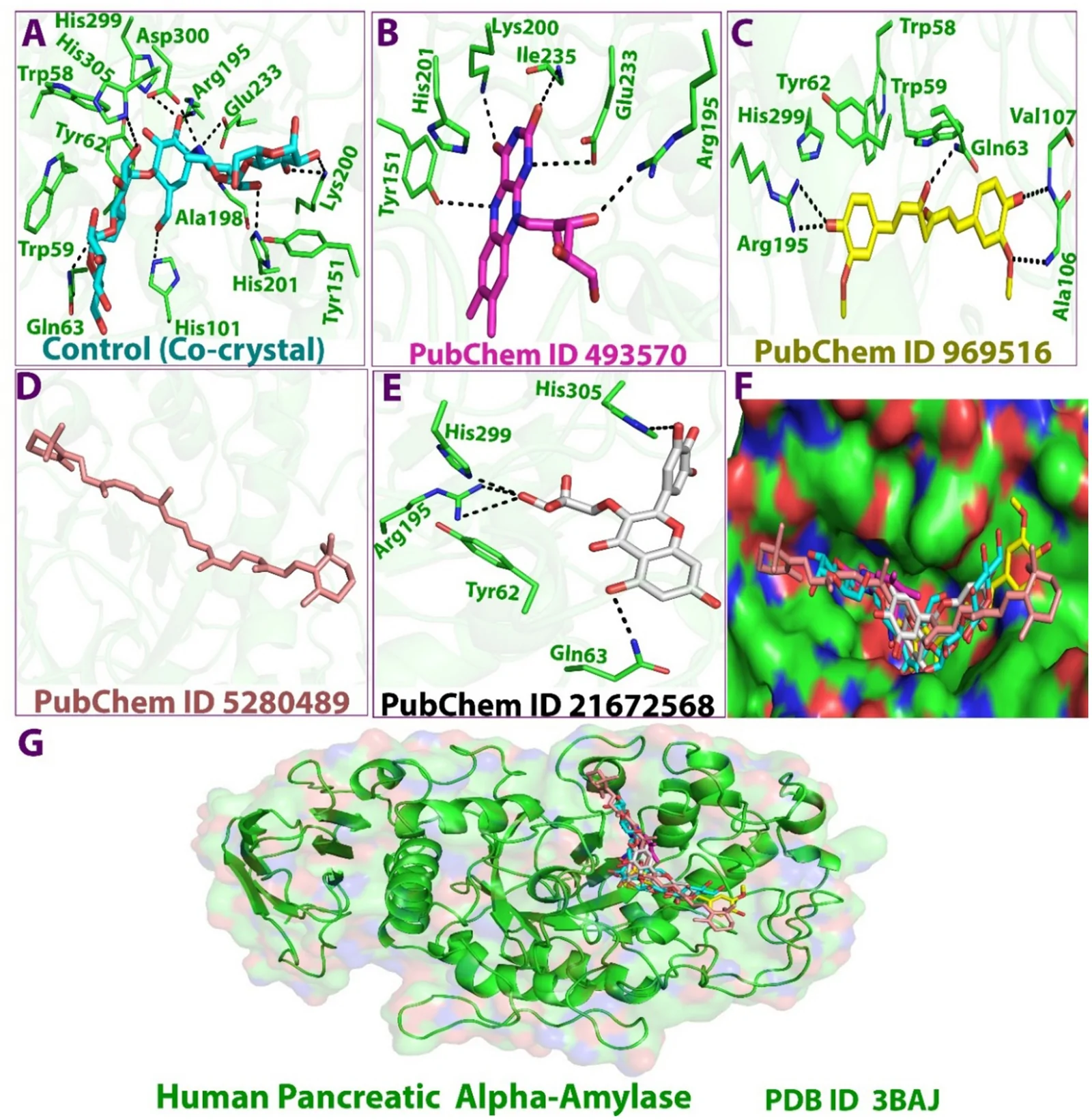
Three-dimensional (3D) visualization of molecular docking interactions between *Centaurea pungens* metabolites and human pancreatic α-amylase (PDB ID: 3BAJ). **(A)** Control (Co-crystal); **(B)** PubChem ID 493570; **(C)** PubChem ID 969516; **(D)** PubChem ID 5280489; **(E)** PubChem ID 21672568; **(F)** Surface representation overlay; **(G)** Overall structure of human pancreatic alpha-amylase.

**Figure 4:**
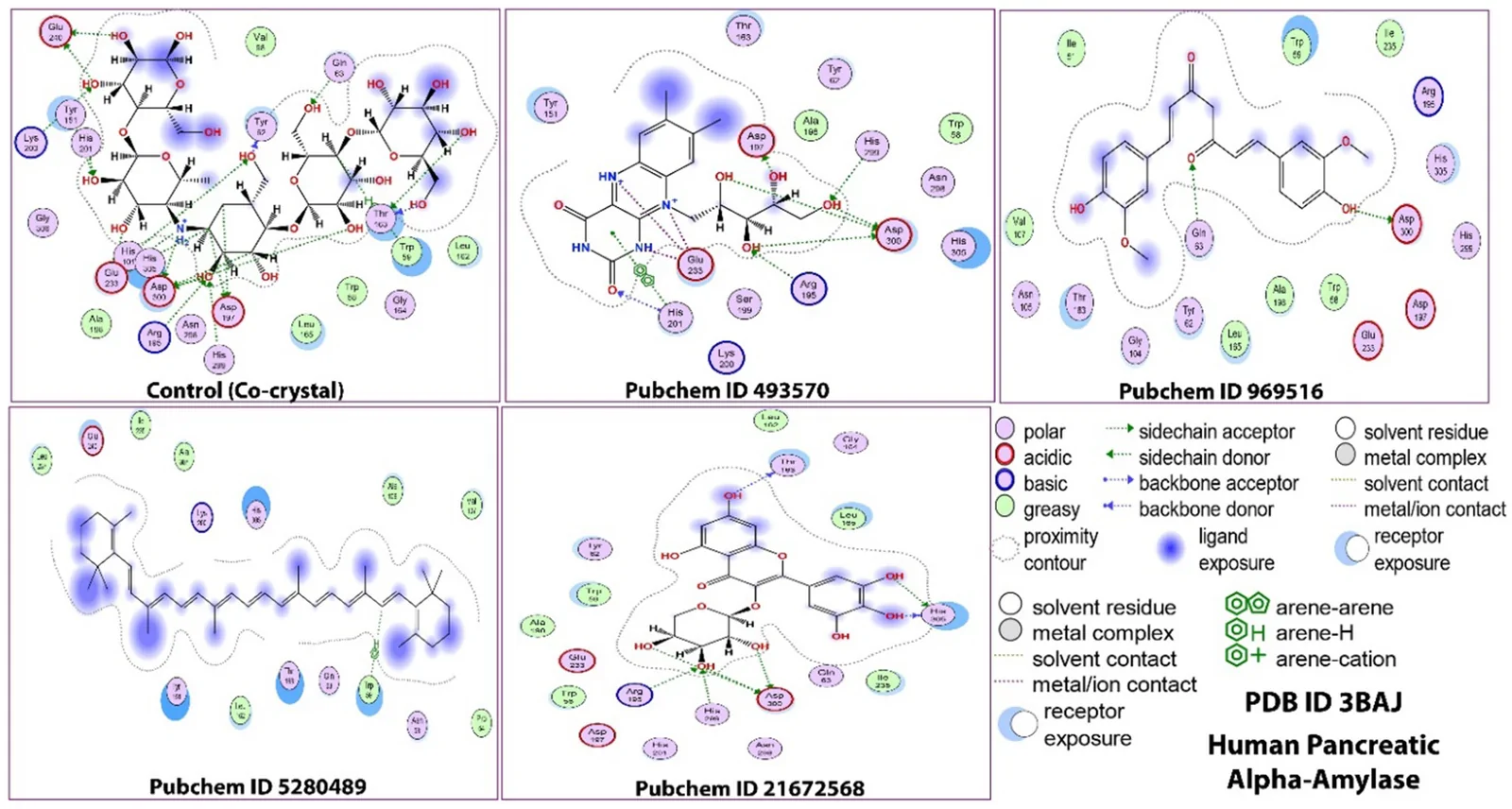
Two-dimensional (2D) protein-ligand interaction diagrams displaying the chemical bonding environment within the HPA active site.

Human Pancreatic α-Amylase (HPA; PDB ID: 3BAJ) features a V-shaped catalytic cleft critical for hydrolyzing α-1,4-glycosidic bonds (Table 2). Molecular docking positioned several *C. pungens* metabolites within this primary active site, overlapping the co-crystal ligand. Among the tested compounds, Myricetin 3-arabinoside (PubChem ID 21672568) displayed high specificity, forming hydrogen bonds with Arg195, His299, and His305. These residues are essential for stabilizing inhibitors and blocking substrate access (29–31). In contrast, the co-crystal ligand interacted mainly with Asp197, Glu233, and Asp300, forming a dense network of hydrogen bonds. Riboflavin and β-carotene displayed fewer interactions, with weaker hydrogen bonding and hydrophobic contacts (32). The 2D and 3D interaction maps predicted that Myricetin 3-arabinoside exhibited superior surface complementarity within the active site (Figures 3, 4). Its interactions with key catalytic residues suggest potential for high inhibitory activity. These docking results highlight the compound's ability to efficiently occupy the active site, mimicking the co-crystal ligand. This strong binding orientation establishes Myricetin 3-arabinoside as a primary candidate for further molecular dynamics evaluation.

**Table 2:** Detailed molecular docking interactions of HPA (3BAJ) with selected inhibitors.

Complex	Ligand atom	Residue	Interaction type	Distance (Å)	Energy (kcal/mol)
Control	N4C	Glu233	H-bond (Ionic)	2.75	−18.5
	O3G	Glu240	H-bond	2.68	−4.4
	O2H	Asp300	H-bond	2.60	−3.2
	O7H	His101	H-bond	2.80	−3.0
Riboflavin	N3	Glu233	Ionic	2.91	−5.1
	O2	Arg195	H-bond	2.79	−4.1
	O4	Asp300	H-bond	2.68	−3.9
	O3	Asp197	H-bond	2.78	−3.7
Curcumin	O5	Gln63	H-bond	2.88	−4.6
	O3	Asp300	H-bond	2.57	−4.0
β-carotene	C22	Trp59	H-pi	4.31	−0.6
Myricetin 3-arabinoside	O4	Asp300	H-bond	2.64	−4.2
	O3	Asp300	H-bond	2.61	−3.8
	O10	His305	H-bond	2.78	−3.0
	O9	Thr163	H-bond	2.63	−3.0
	O3	Arg195	H-bond	2.87	-

It should be emphasized that molecular docking and molecular dynamics simulations provide mechanistic hypotheses regarding protein–ligand interactions rather than direct experimental evidence of enzyme inhibition. Therefore, these computational findings should be interpreted as complementary to the *in vitro* α-amylase inhibition assay and primarily serve to identify phytochemicals that may contribute to the observed biological activity.

Furthermore, given that the present study lacks absolute quantitative data for the individual compounds identified by UPLC-ESI-MS/MS, any attribution of the observed α-amylase inhibitory, antioxidant, or antimicrobial activities to specific metabolites should be interpreted with caution. The biological effects reported herein reflect the collective action of the crude extract, and while computational and correlative evidence suggests that certain compounds, particularly myricetin 3-arabinoside, may contribute to these activities, definitive compound-specific efficacy cannot be established without quantitative phytochemical validation and isolation-based bioassays. The synergistic, additive, or antagonistic interactions among the multiple constituents of the extract further complicate such attributions.

#### 3.3.2 RMSD analysis of complex stability

**Figure 5:**
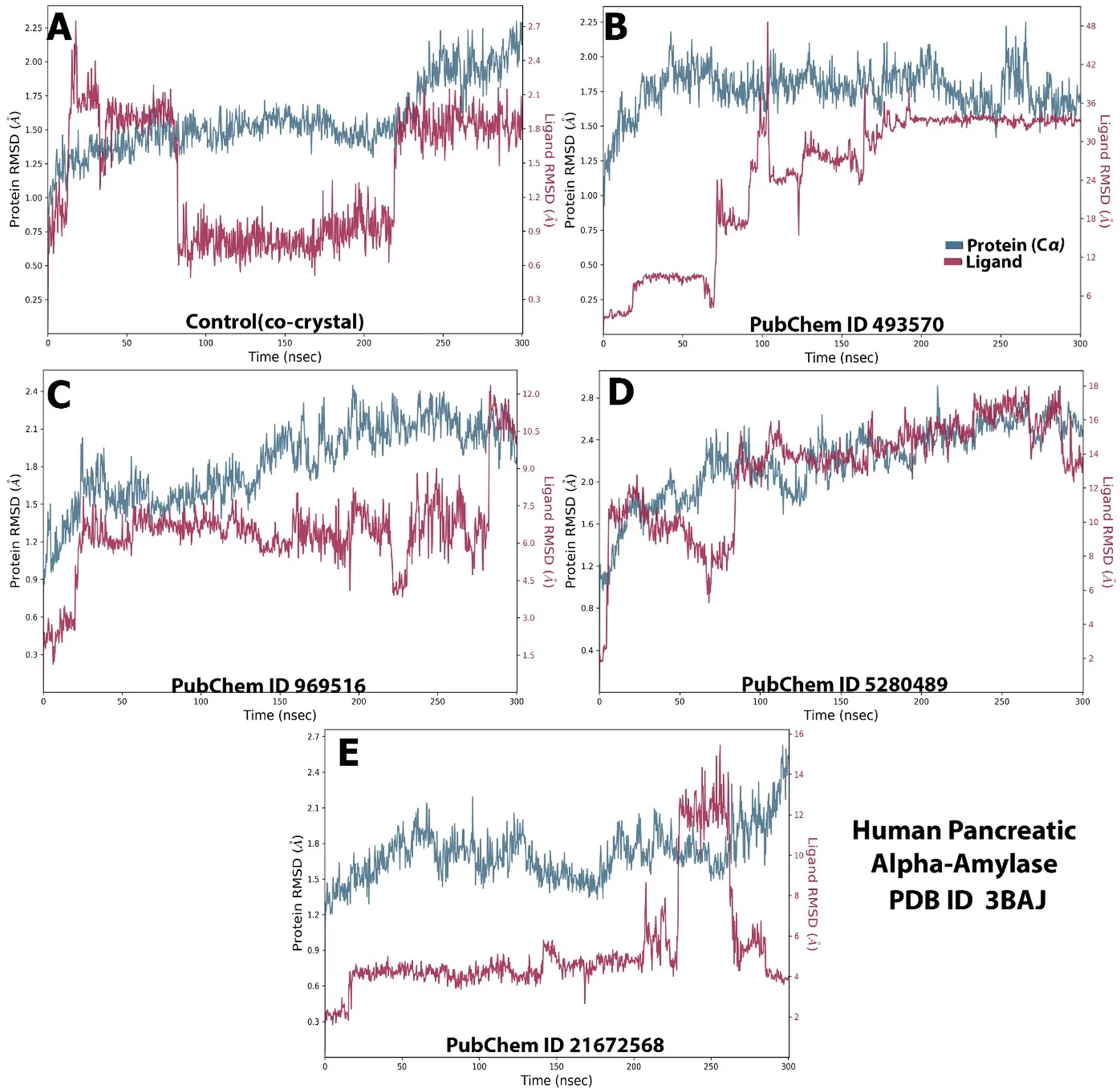
Molecular dynamics simulation and stability analysis of human pancreatic α-amylase (PDB ID: 3BAJ) complexed with potential small molecule inhibitors. **(A)** Control (Co-crystal); **(B)** PubChem ID 493570; **(C)** PubChem ID 969516; **(D)** PubChem ID 5280489; **(E)** PubChem ID 21672568.

To assess dynamic stability, 300 ns molecular dynamics (MD) simulations were performed for HPA complexes. The protein backbone remained stable across all systems, with RMSD values ranging from 1.25 to 2.4 Å, indicating preserved structural integrity (Figure 5). Ligand RMSD revealed marked differences in binding persistence. Myricetin 3-arabinoside maintained a stable profile for the first 200 ns. After a transient peak near 250 ns, the ligand re-established its orientation and stabilized around 4.0 Å. This demonstrates sustained occupancy of the catalytic site and retention of key interactions. In contrast, Riboflavin showed extreme fluctuations exceeding 30 Å, reflecting loss of binding and dissociation from the active site. Curcumin and β-carotene displayed intermediate RMSD behavior, indicating partial stability. These results indicate that hydrogen-bonded flavonoid glycosides, particularly Myricetin 3-arabinoside, offer superior dynamic stability in the HPA pocket compared with small molecules or carotenoids. RMSD analysis confirms that only compounds capable of maintaining hydrogen bonding and hydrophobic complementarity retain long-term stability, consistent with the high binding energies predicted in docking studies. These findings emphasize the importance of both spatial fit and chemical interactions for effective enzyme inhibition.

The relatively stable RMSD values observed throughout the simulation indicate that the ligand remained associated with the enzyme without major conformational disruption. Together with the favorable MM-GBSA binding energies, these findings support the structural stability of the predicted complex under simulated physiological conditions.

#### 3.3.3 Local flexibility and residue fluctuations

**Figure 6:**
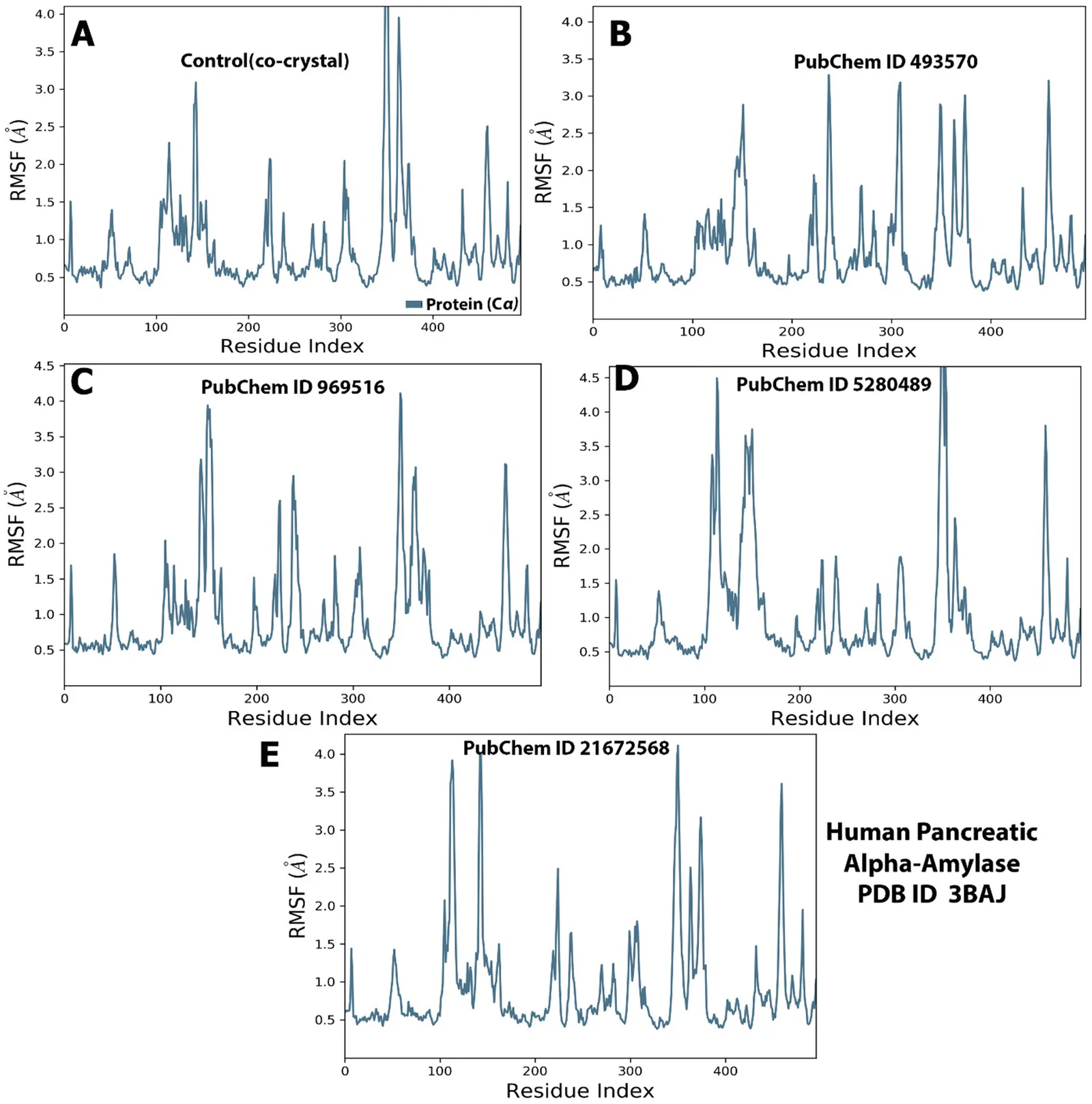
Comparative root mean square fluctuation (RMSF) mapping of human pancreatic α-amylase residues upon interaction with selected ligands. **(A)** Control (Co-crystal); **(B)** PubChem ID 493570; **(C)** PubChem ID 969516; **(D)** PubChem ID 5280489; **(E)** PubChem ID 21672568.

Residue-level flexibility was analyzed using Root Mean Square Fluctuation (RMSF). Residues forming the catalytic triad (Asp197, Glu233, Asp300) exhibited minimal fluctuations (< 1.5 Å) when bound to Myricetin 3-arabinoside, comparable to the co-crystal ligand (Figure 6). This demonstrates that the ligand effectively stabilizes the enzyme's functional core. Hydrogen bonds with Asp197 and His305 act as anchors, “locking” the enzyme into an inhibited conformation. Riboflavin induced higher fluctuations (>3.0 Å) in residues 300–400, reflecting unstable binding and increased structural noise. Curcumin and β-carotene showed moderate RMSF values, suggesting partial stabilization. Analysis of other secondary structure regions confirmed that only ligands forming persistent hydrogen bonds and hydrophobic contacts maintain protein rigidity. These results support RMSD findings, showing that Myricetin 3-arabinoside preserves enzymatic integrity while preventing substrate access. The stable RMSF profile correlates with high inhibitory potential and suggests favorable thermodynamic interactions. These observations are consistent with literature reporting flavonoid glycosides as potent α-amylase inhibitors due to their ability to stabilize active-site residues and prevent conformational fluctuations.

#### 3.3.4 Quantitative protein-ligand interaction fingerprinting

**Figure 7:**
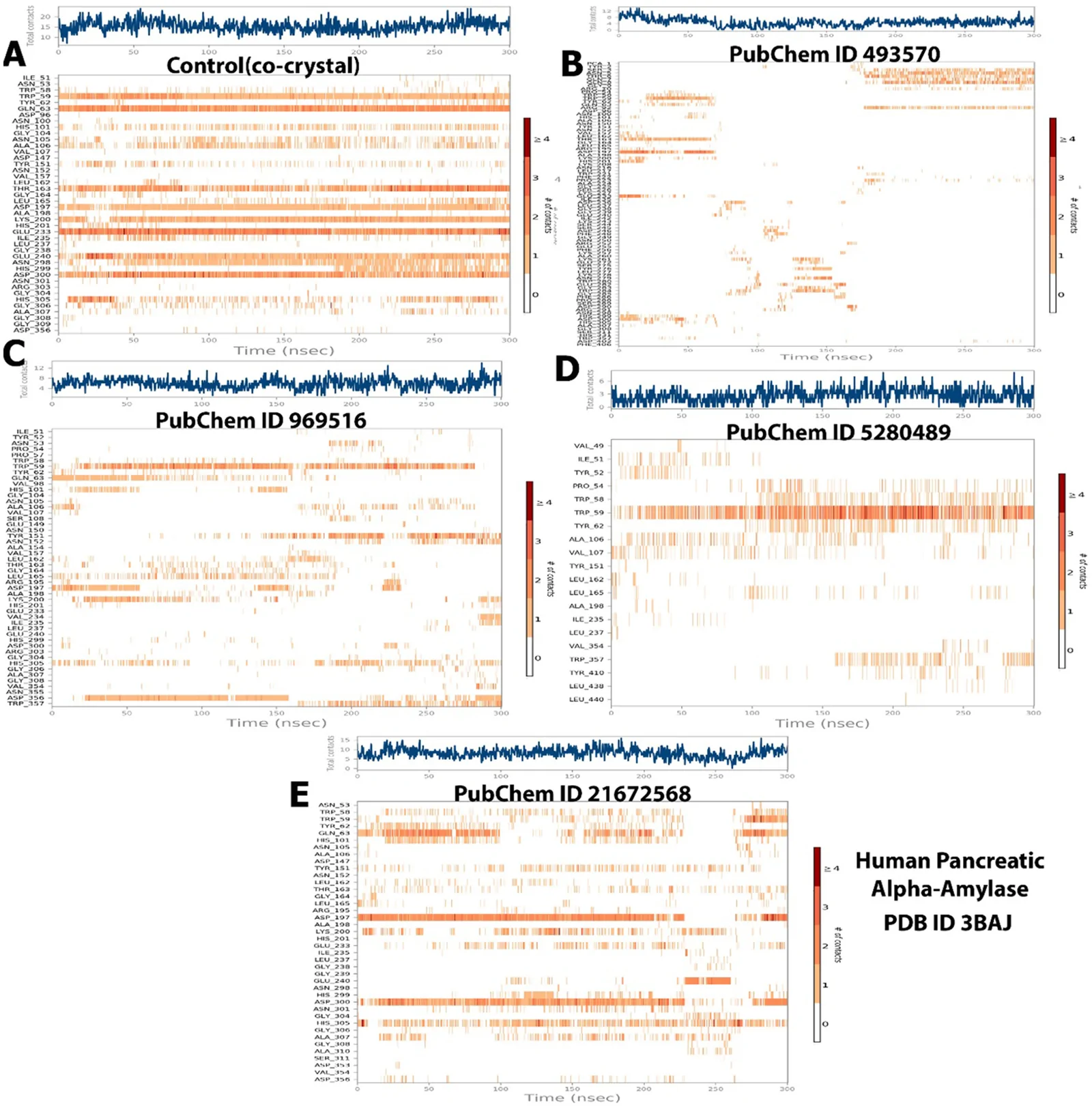
Time-resolved mapping of residue-level interactions and contact stability between human pancreatic α-amylase and potential lead compounds. **(A)** Control (Co-crystal); **(B)** PubChem ID 493570; **(C)** PubChem ID 969516; **(D)** PubChem ID 5280489; **(E)** PubChem ID 21672568.

Interaction fingerprinting over 300 ns revealed the chemical nature and persistence of contacts. Myricetin 3-arabinoside exhibited the highest interaction fraction at Asp197 (~1.4), maintained via hydrogen bonds and water-mediated bridges for almost the entire simulation (Figure 7). Hydrogen bonds ranged between 3 and 7 per frame, without zero-bond gaps, indicating stable occupancy. β-Carotene interacted mainly through hydrophobic stacking with Trp59, while Riboflavin displayed intermittent bonding, explaining its unstable trajectory. Water bridges and ionic interactions contributed minimally to carotenoids and small phenolics but supported flavonoid glycosides. These data emphasize that both the number and persistence of interactions are critical for inhibitor effectiveness. Myricetin 3-arabinoside closely mimics the co-crystal ligand, targeting key residues involved in catalysis. The high density of hydrogen bonds, combined with hydrophobic and polar interactions, demonstrates a strong complementarity to the enzyme's binding pocket. These results reinforce that flavonoid glycosides are better candidates than carotenoids or riboflavin for selective, sustained HPA inhibition.

#### 3.3.5 Radius of gyration and structural compactness

**Figure 8:**
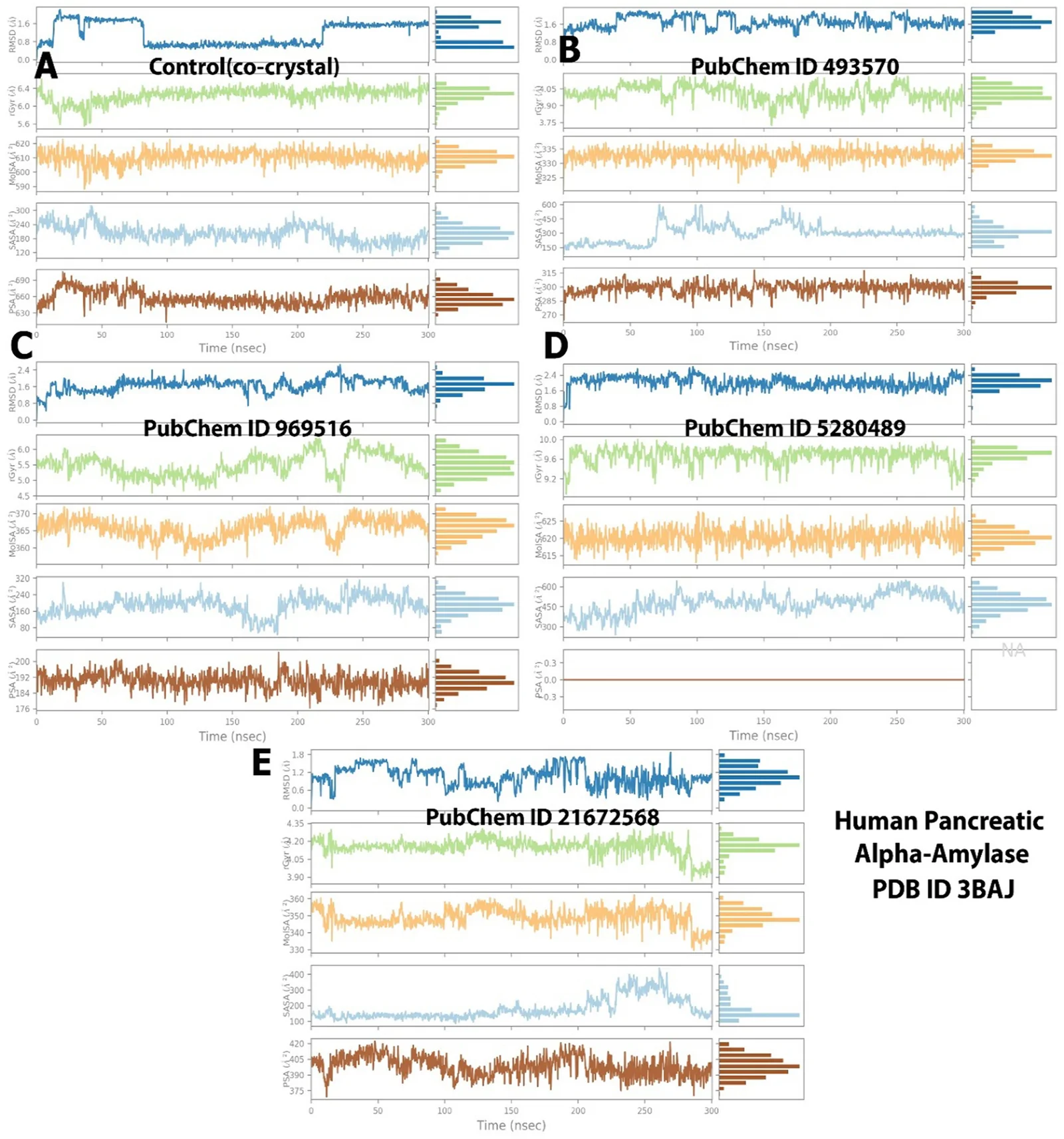
Structural dynamics and residue-level interactions of human pancreatic α-amylase with high-affinity ligands: integrating RMSD, RMSF, and contact mapping. **(A)** Control (Co-crystal); **(B)** PubChem ID 493570; **(C)** PubChem ID 969516; **(D)** PubChem ID 5280489; **(E)** PubChem ID 21672568.

Radius of gyration (Rg) was monitored to evaluate protein compactness. Across all complexes, Rg fluctuated narrowly between 22.8 and 23.6 Å, confirming that HPA maintained its globular fold during MD simulations (Figure 8). Myricetin 3-arabinoside maintained an Rg nearly identical to the co-crystal control, indicating minimal structural perturbation. Riboflavin and β-carotene caused minor transient expansions, suggesting partial destabilization. No ligand-induced sustained unfolding or denaturation. This structural stability aligns with RMSD and RMSF results, demonstrating that Myricetin 3-arabinoside preserves the enzyme's tertiary structure while effectively occupying the active site. The compactness profile confirms that high-affinity ligands can inhibit the enzyme without causing structural damage, supporting their suitability for further *in vitro* and *in vivo* studies.

#### 3.3.6 Binding free energy validation via MM-GBSA

MM-GBSA calculations quantified ligand binding affinity. Myricetin 3-arabinoside showed ΔG_bind_ = −46.22 kcal/mol, with strong van der Waals (−35.44 kcal/mol) and hydrogen bond (−8.37 kcal/mol) contributions. The co-crystal ligand had ΔG_bind_ = −78.64 kcal/mol, confirming strong reference binding. Riboflavin exhibited the weakest binding (−33.45 kcal/mol), consistent with its poor RMSD and RMSF behavior. Curcumin and β-carotene displayed moderate affinities (−60.61 and −60.93 kcal/mol). These results integrate with docking and MD analyses, highlighting Myricetin 3-arabinoside as the most promising HPA inhibitor. Persistent hydrogen bonding and surface complementarity underpin its high-affinity, dynamic stability, and potential as a therapeutic candidate (Table 3).

**Table 3:** Binding energies (MMGBSA) of the complexes of human pancreatic α-amylase and the selected compounds.

Compounds	MMGBSA dG Bind (Kcal/mol)	MMGBSA dG Bind Coulomb (Kcal/mol)	MMGBSA dG Bind Covalent (Kcal/mol)	MMGBSA dG Bind Hbond (Kcal/mol)	MMGBSA dG Bind Lipo (Kcal/mol)	MMGBSA dG Bind Solv GB (Kcal/mol)	MMGBSA dG Bind vdW (Kcal/mol)
Control (co-crystal)	−78.63774	−44.093811	8.30091	−7.28501	−28.57887	59.27397	−66.25493
Riboflavin	−33.45358	−21.09818	2.51197	−4.58551	−10.2552	42.64512	−41.15254
Curcumin	−60.61235	−38.9779	3.35342	−2.05802	−18.62924	38.84949	−40.86203
B-carotene	−60.92834	−2.1992	5.97966	0	−33.44492	23.06064	−54.32452
Myricetin 3-arabinoside	−46.22249	−31.96111	3.55023	−4.65053	−8.365990	34.026790	−35.441409

*Centaurea* species exhibit promising anti-diabetic properties, primarily through inhibition of carbohydrate-digesting enzymes and modulation of glucose homeostasis, aligning with traditional uses in folk medicine (33).

Extracts from *C. urvillei, C. virgata*, and *C. calcitrapa* demonstrate potent α-glucosidase and α-amylase inhibition, often surpassing acarbose in methanol and ethyl acetate fractions, due to flavonoids like cosmosiin, cynaroside, and nepetin. These compounds bind enzyme active sites, delaying starch and disaccharide hydrolysis to reduce postprandial hyperglycemia, as confirmed by LC-MS/MS profiling and molecular docking. Such activity mirrors broader *Centaurea* phenolic profiles, where glycosylated flavonoids enhance solubility and binding affinity (34, 35).

Phenolics and flavonoids underpin these activities, with nepetin showing high α-amylase affinity in docking studies. Reviews confirm consistent efficacy across more than 20 *Centaurea* spp., supporting development of herbal remedies without clinical trials to date. For *C. pungens*, the detected curcumin and quercetin glycosides suggest similar potential, warranting targeted isolation for type 2 diabetes management (33, 35).

### 3.4 *In vitro* α-amylase inhibitory activity

**Figure 9:**
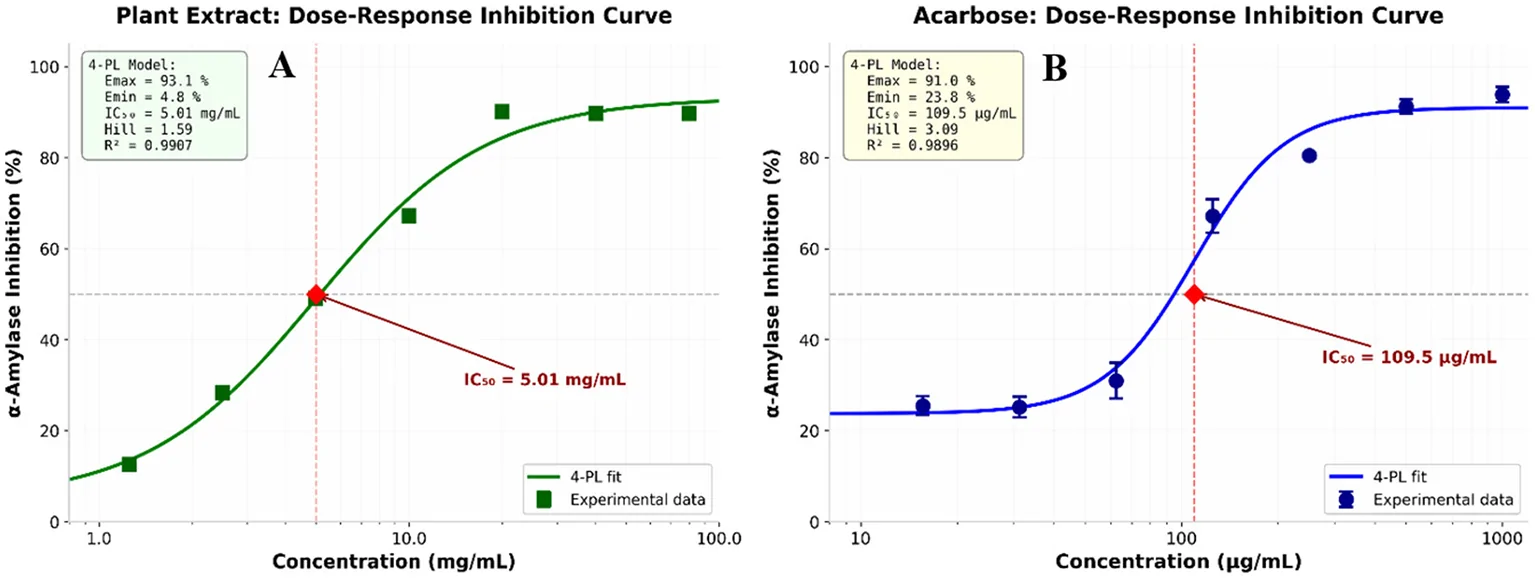
Dose-response inhibition curves against porcine pancreatic α-amylase. **(A)** *C. pungens* ethanolic extract; **(B)** Acarbose (positive control).

To experimentally validate the computational predictions, the α-amylase inhibitory activity of the *C. pungens* ethanolic extract was evaluated using a starch-iodine colorimetric assay, with acarbose as the reference inhibitor. The extract exhibited concentration-dependent inhibition of porcine pancreatic α-amylase, with inhibition increasing from 12.49% ± 0.34% at 1.25 mg/mL to a plateau of approximately 90% at concentrations ≥20 mg/mL (89.76% ± 0.53%, 89.75% ± 0.53%, and 90.01% ± 0.51% at 80, 40, and 20 mg/mL, respectively) (Figure 9A). Nonlinear regression analysis yielded an IC50 of 5.01 ± 1.01 mg/mL (*R*^2^ = 0.9907). On the other hand, the reference inhibitor acarbose demonstrated a steep dose-response relationship, with inhibition ranging from 25.50% ± 2.11% at 15.63 μg/mL to 93.84% ± 1.66% at 1,000 μg/mL (Figure 9B), with an IC50 of 109.52 ± 10.99 μg/mL (*R*^2^ = 0.9896).

**Figure 10:**
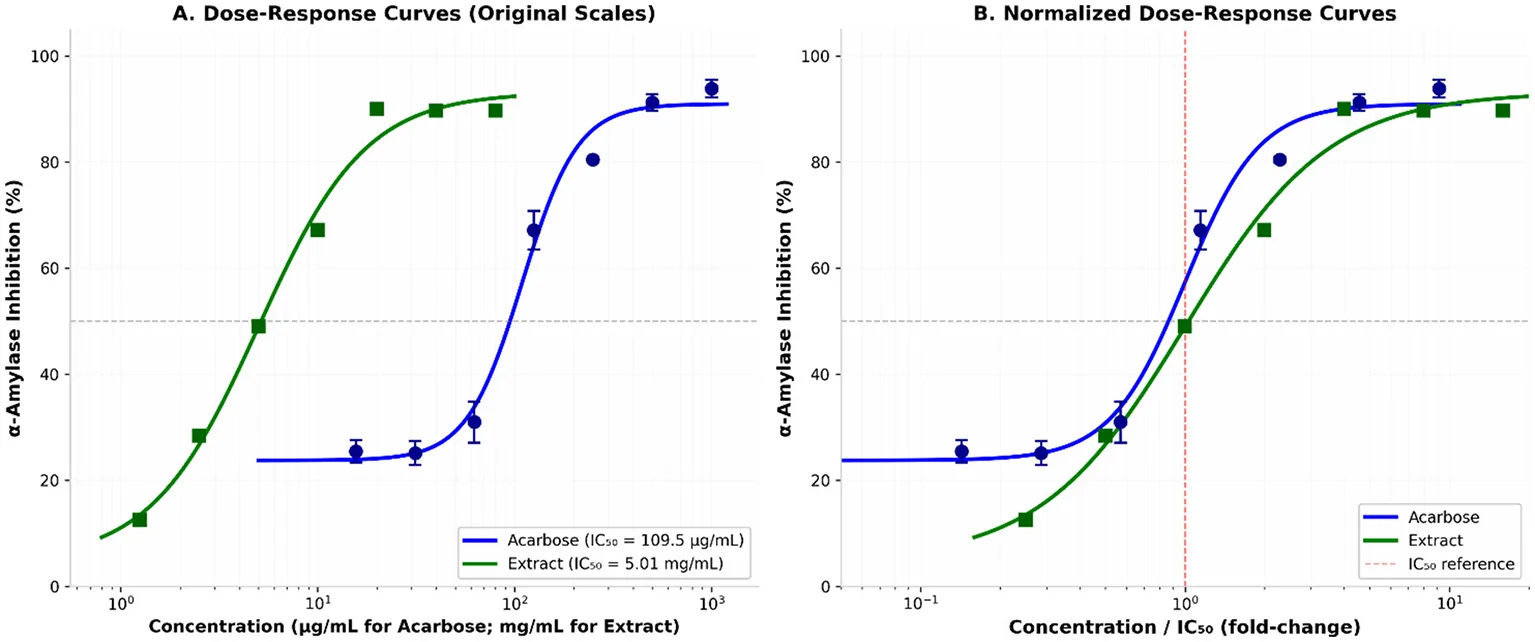
Comparative analysis of dose-response relationships. **(A)** Both acarbose and *C. pungens* extract were plotted on their respective concentration scales (μg/mL for acarbose; mg/mL for extract). **(B)** Normalized curves with concentrations expressed as fold-change relative to the respective IC_50_ values, enabling direct comparison of curve shape and dynamic range.

The *C. pungens* extract was less potent than acarbose. However, the extract achieved a comparable maximal inhibitory effect (Emax = 93% vs. 91% for acarbose), indicating that the extract contains bioactive constituents capable of near-complete enzyme inhibition. The normalized dose-response curves (Figure 10) revealed that the extract exhibits a broader dynamic range and a more gradual dose-response transition compared to acarbose, consistent with the polyphenolic cocktail effect of crude plant matrices.

The *C. pungens* extract exhibited concentration-dependent α-amylase inhibition with an IC50 of 5.01 mg/mL, confirming that the phytochemical constituents identified by UPLC-ESI-MS/MS, particularly the abundant flavonoid glycosides, indeed possess measurable enzyme inhibitory activity. The experimental IC50 aligns with the molecular docking and MD simulation results, which identified myricetin 3-arabinoside as the compound with the highest binding affinity (ΔG_bind = −46.22 kcal/mol) and greatest dynamic stability (RMSD stabilizing at ~4.0 Å after 200 ns). This correlation between *in silico* prediction and *in vitro* validation strengthens the mechanistic interpretation that flavonoid glycosides, especially myricetin derivatives, are the primary drivers of the observed α-amylase inhibition.

The extract's IC50 is consistent with the range reported for other *Centaurea* species evaluated under comparable *in vitro* conditions. For instance, methanol extracts of *C. calcitrapa* and *C. urvillei* demonstrated potent α-amylase and α-glucosidase inhibition, often surpassing acarbose in bioassay-guided fractions enriched in flavonoids such as cosmosiin, cynaroside, and nepetin (35, 36). The difference between the *C. pungens* extract and acarbose on a mass basis is expected for a crude extract, given that only a fraction of the total mass corresponds to bioactive flavonoids. Notably, the extract achieved a comparable maximal inhibitory effect (~93% vs. ~91% for acarbose), suggesting that at sufficiently high concentrations, the combined action of multiple polyphenolic constituents can achieve near-complete enzyme inhibition. This multitarget inhibition profile may confer therapeutic advantages, including reduced risk of resistance development and broader anti-hyperglycemic effects.

The extract showed that its inhibitory action likely involved multiple compounds with distinct binding modes, rather than a single high-affinity competitive inhibitor. This is consistent with the polyphenolic complexity revealed by UPLC-ESI-MS/MS, where myricetin 3-arabinoside, quercetin-3-glucoside, rutin, and other flavonoid glycosides may act synergistically or additively to inhibit α-amylase. Such polypharmacological behavior is characteristic of crude plant extracts and has been documented across numerous medicinal plants, where the combined action of multiple phenolic compounds often exceeds the activity of any single isolated constituent (37, 38).

### 3.5 Antioxidant activities

**Figure 11:**
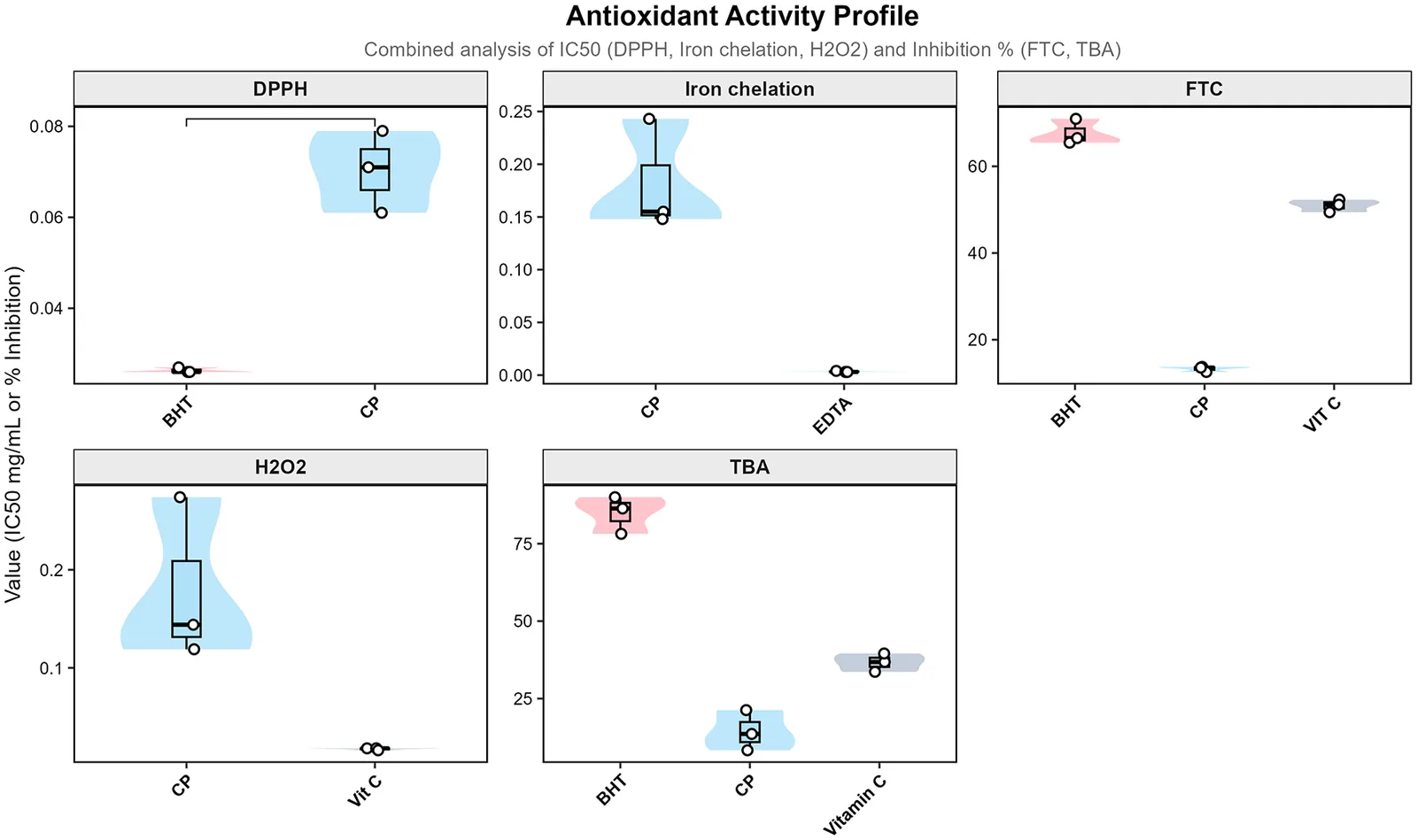
Comparative antioxidant activity of *C. pungens* ethanolic extract and standard controls.

The antioxidant potential of *C. pungens* extract was evaluated using a multi-mechanistic approach, including radical scavenging (DPPH, H_2_O_2_), metal chelation, and lipid peroxidation inhibition (FTC and TBA). The results are summarized in Table 4 and Figure 11. In the DPPH assay, the extract showed a notable IC50 of 0.0706 ± 0.006 mg/mL, reflecting strong hydrogen-donating capacity. Although this value is higher than that of the synthetic antioxidant BHT (IC50 = 0.0260 ± 0.0006 mg/mL), it indicates substantial polyphenolic and flavonoid-mediated radical stabilization. The H_2_O_2_ scavenging assay revealed an IC50 of 0.1789 ± 0.083 mg/mL, demonstrating the extract's ability to neutralize hydrogen peroxide and indirectly prevent hydroxyl radical formation via the Fenton reaction. This suggests potential intracellular protection against oxidative stress.

**Table 4:** Antioxidant and radical scavenging activity of *C. pungens* extract.

Method	*C. pungens* extract	BHT	Vitamin C	EDTA
DPPH (IC_50_ mg/mL)	0.0706 ± 0.006	0.0260 ± 0.0006	-	-
H_2_O_2_ (IC_50_ mg/mL)	0.1789 ± 0.083	-	0.0173 ± 0.001	-
Iron chelation (IC_50_ mg/mL)	0.1821 ± 0.053	-	-	0.0034 ± 0.0006
FTC inhibition (%)	13.29 ± 0.63	67.65 ± 2.91	50.96 ± 1.45	-
TBA inhibition (%)	14.39 ± 6.52	84.85 ± 6.05	36.67 ± 2.96	-

Metal chelation is a key secondary antioxidant mechanism that prevents the initiation of radical chain reactions. The extract showed moderate iron-chelating activity (IC50 = 0.1821 ± 0.053 mg/mL), highlighting its preventive antioxidant properties. Regarding lipid peroxidation, the extract inhibited hydroperoxide formation by 13.29% ± 0.63% in the FTC assay and malondialdehyde (MDA) accumulation by 14.39% ± 6.52% in the TBA assay. These consistent values indicate that *C. pungens* maintains antioxidant activity throughout both early and late stages of lipid oxidation. While inhibition is significantly lower than BHT (67%−85%), the results confirm that polar radical scavengers in the extract dominate its antioxidant profile, whereas lipophilic components may be less concentrated.

The antioxidant activity observed in *C. pungens* is consistent with the high phenolic content detected in this study. Numerous reports have demonstrated that antioxidant capacity in *Centaurea* species correlates strongly with total phenolic and flavonoid content (6, 7, 10). For example, *C. bingoelensis* exhibited strong reducing power linked to its phenolic richness. Similarly, *C. parviflora* showed notable radical scavenging activity. The antioxidant mechanisms are mainly attributed to hydrogen donation, metal chelation, and inhibition of lipid peroxidation. These mechanisms were also confirmed experimentally in the present study. The moderate-to-strong antioxidant activity of *C. pungens* supports the functional role of its polyphenolic constituents. This finding aligns with previous literature and validates its potential as a natural antioxidant source.

The stronger radical scavenging activity compared with metal-chelating activity probably reflects the predominance of hydroxylated phenolic compounds capable of donating hydrogen atoms to neutralize free radicals (39, 40). In contrast, effective metal chelation generally requires specific structural arrangements that may be less abundant in the extract.

While the strong correlation between total phenolic content and antioxidant capacity supports the general involvement of polyphenolic constituents, the absence of quantitative data for individual compounds precludes identifying which specific metabolites are primarily responsible for each antioxidant mechanism (e.g., radical scavenging vs. metal chelation vs. lipid peroxidation inhibition). Future studies employing compound-specific quantification and fractionation-based activity profiling are needed to resolve these contributions.

### 3.6 Antibacterial activity

**Figure 12:**
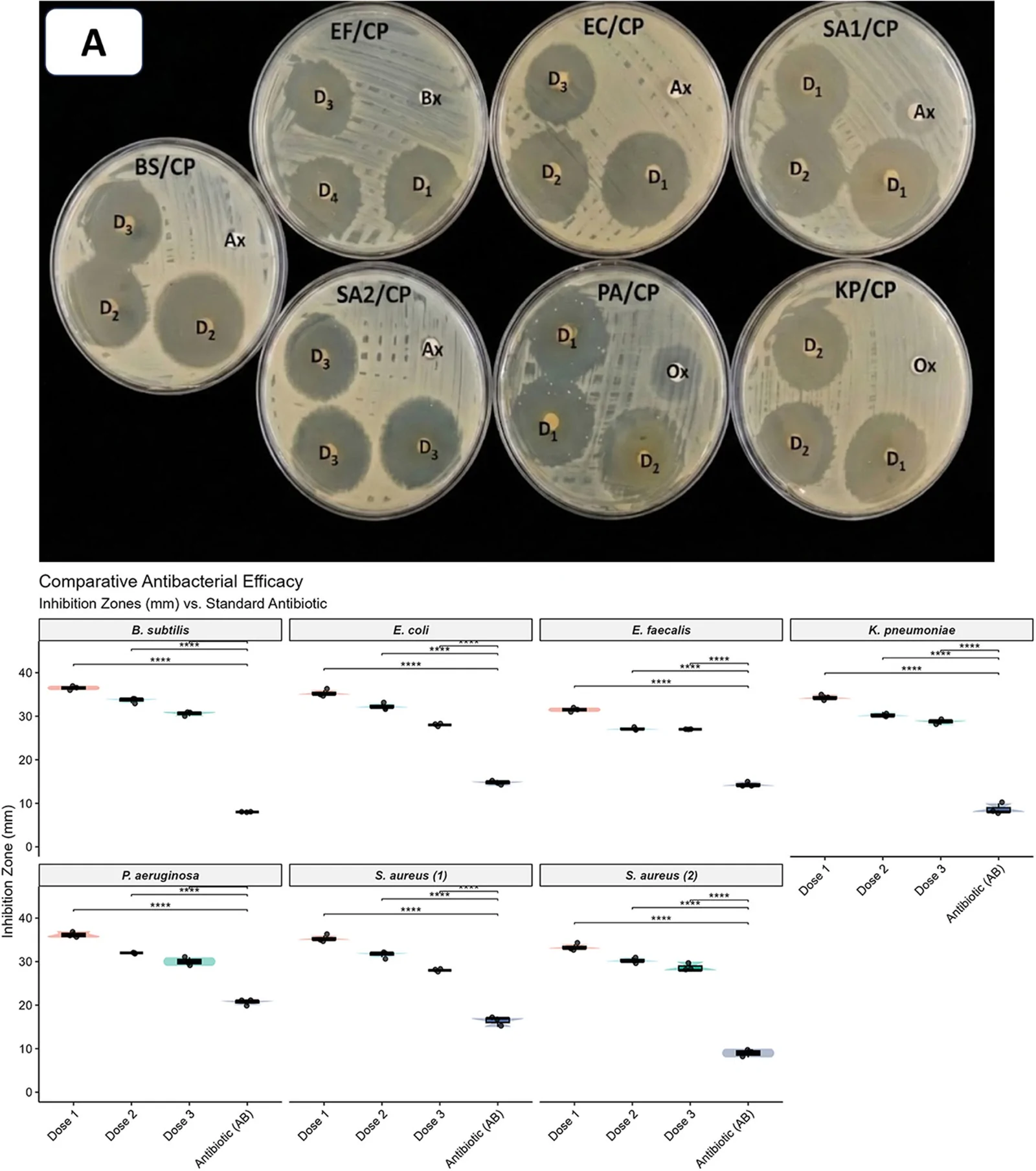
Antibacterial activity of *C. pungens* extracts. **(A)** Visual representation of inhibition zones obtained via disc diffusion. **(B)** Quantitative analysis of inhibition zone diameters across three concentration levels (D1–D3) compared to an antibiotic control (AB).

The antibacterial activity of *C. pungens* extract was evaluated against Gram-positive and Gram-negative bacteria using the agar disc diffusion method. The extract exhibited a clear dose-dependent effect, with inhibition zone diameters decreasing from D1 to D3 across all strains (Figure 12). At the highest concentration (D1), inhibition zones ranged from approximately 32 to 35 mm. At D2, values decreased to 28–33 mm, while at D3 they ranged from 27 to 30 mm. Despite this reduction, the extract maintained strong antibacterial activity at all tested concentrations. Notably, the inhibition zones produced by *C. pungens* at D1 were significantly higher (*p* < 0.01) than those of the antibiotic control (8–21 mm) for several strains. This effect was particularly pronounced against *Bacillus subtilis* and *Pseudomonas aeruginosa*, where the extract outperformed standard antibiotics. The high sensitivity of *P. aeruginosa* is especially important, as this species is typically associated with strong intrinsic resistance. *Staphylococcus aureus* strains also showed marked susceptibility, indicating broad-spectrum activity. In contrast, *Enterococcus faecalis* exhibited lower inhibition zones, suggesting reduced sensitivity, possibly due to differences in cell wall composition and permeability. The *C. pungens* demonstrated strong, broad-spectrum antibacterial activity with statistically significant effects at higher concentrations. The large inhibition zones, particularly against resistant strains, highlight its potential as a natural antimicrobial agent. These findings are consistent with previous reports linking phenolic-rich plant extracts to membrane disruption, enzyme inhibition, and oxidative damage in bacterial cells (41).

Antibacterial activity was pronounced, with inhibition zones up to 36.5 mm, significantly exceeding the antibiotic control (8–21 mm; ^****^p < 0.0001). The antibacterial results demonstrate that *C. pungens* exhibits strong, dose-dependent activity against both Gram-positive and Gram-negative bacteria. The inhibition zones were significantly higher than the antibiotic control in several cases (*p* < 0.0001). This highlights the potency of the extract, particularly against *B. subtilis* and *P. aeruginosa*. Similar antibacterial effects have been reported for *C. bingoelensis* and *C. parviflora* (6, 10). In many *Centaurea* species, antimicrobial activity is linked to sesquiterpene lactones. However, these compounds were not detected in *C. pungens*. Therefore, the observed antibacterial effect is likely driven by flavonoids and phenolic acids, which are known to disrupt bacterial membranes, inhibit enzymes, and induce oxidative stress. This suggests an alternative mechanism of action compared to lactone-rich species. The results confirm that *C. pungens* is a promising source of natural antibacterial agents.

The pronounced antibacterial activity is likely associated with the presence of quercetin, luteolin, apigenin, ferulic acid, curcumin, and myricetin derivatives, all of which have been widely reported to disrupt bacterial membranes, inhibit nucleic acid synthesis, and interfere with essential bacterial enzymes (42–44). The generally greater susceptibility of Gram-positive bacteria compared with Gram-negative bacteria may be explained by differences in cell envelope architecture. Gram-negative bacteria possess an outer membrane enriched in lipopolysaccharides that restricts the penetration of many polyphenolic compounds, whereas the thicker but more permeable peptidoglycan layer of Gram-positive bacteria offers less resistance to phytochemical diffusion (45, 46).

The antimicrobial activity observed is unlikely to result from a single metabolite. Instead, phenolic acids, flavonoids, carotenoids, and other secondary metabolites probably act through complementary mechanisms, including disruption of membrane integrity, interference with microbial enzymes, induction of oxidative stress, and inhibition of biofilm formation (42, 47).

### 3.7 Antifungal activity

The antifungal activity of *C. pungens* ethanolic extract was further quantified by determining the MIC and MFC against *F. oxysporum, A. alternata*, and *A. solani*. Complete inhibition of visible fungal growth was observed at 75 mg/mL, whereas growth occurred at 50 mg/mL and lower concentrations. Accordingly, the MIC was established at 75 mg/mL for all three fungal species. To determine the fungicidal activity, mycelial plugs from growth-inhibited tubes were transferred onto fresh PDA medium. No fungal regrowth was observed after subculturing from the 75 mg/mL treatment, confirming that this concentration was also fungicidal. Therefore, the MFC was likewise determined to be 75 mg/mL for all tested fungi. The identical MIC and MFC values indicate that the extract exhibited fungicidal activity under the experimental conditions employed.

**Figure 13:**
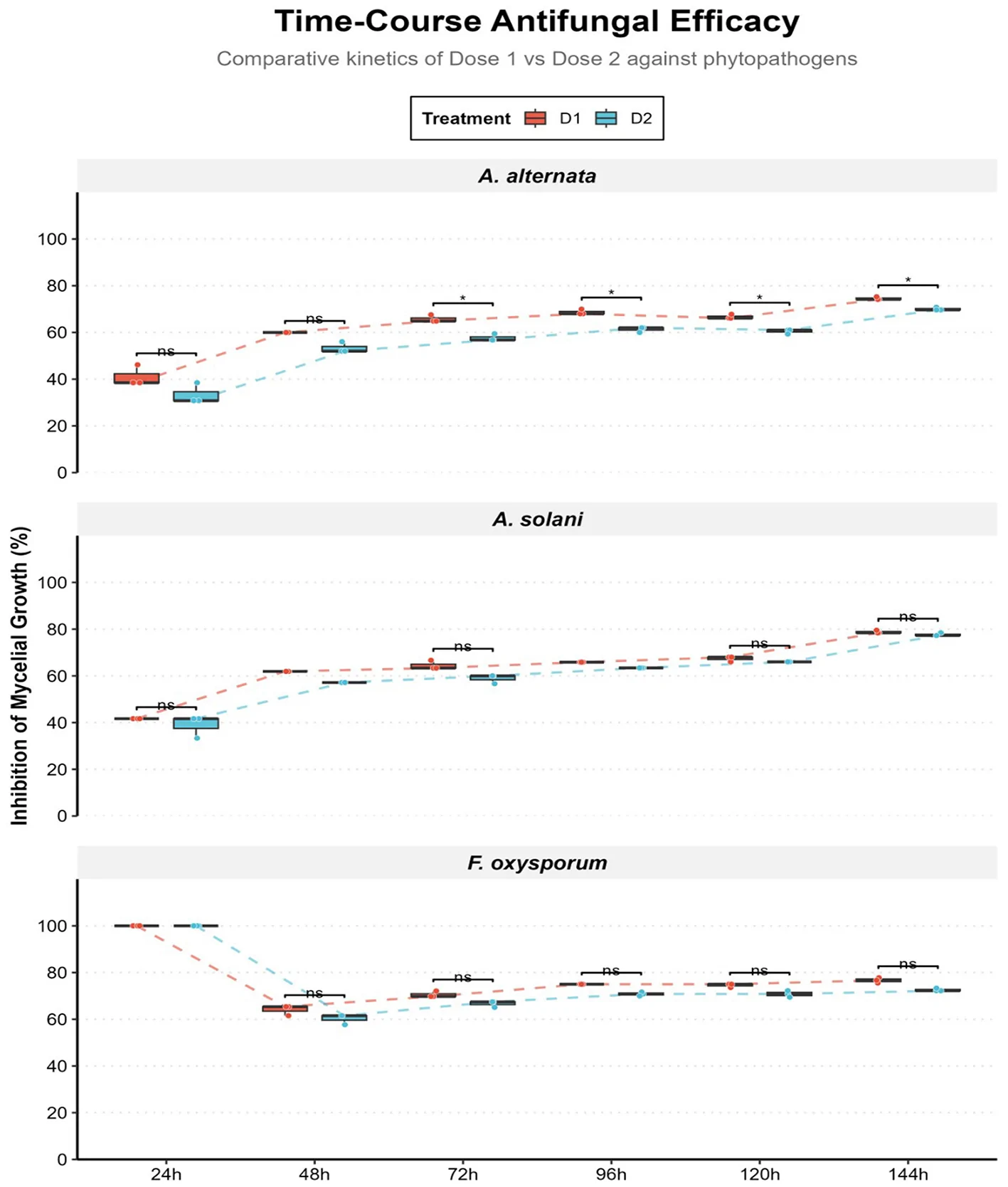
Comparative time-course of antifungal activity of *C. pungens* against *A. alternata, A. solani*, and *F. oxysporum*.

On the other hand, *C. pungens* extract was evaluated against the three fungi over 144 h at two concentrations (D1:100 mg/mL and D2: 75 mg/mL). The extract exhibited time-dependent inhibition of mycelial growth in all tested fungi (Figure 13). For *A. alternata*, D1 showed consistently higher inhibition than D2. The difference became statistically significant at later time points (72–144 h; *p* < 0.05). At 144 h, inhibition reached approximately 75% for D1 and 70% for D2. At earlier stages (24–48 h), differences were minimal, indicating a progressive antifungal effect. This suggests that higher concentrations enhance long-term inhibition efficiency. For *A. solani*, both concentrations produced similar inhibition profiles. No significant differences were observed at any time point (ns). Inhibition increased gradually and reached approximately 78% at 144 h for both doses. This indicates that even lower concentrations are sufficient to achieve maximal antifungal activity against this species. For *F. oxysporum*, both doses induced complete inhibition (100%) at 24 h, representing the most significant result. However, inhibition decreased to 60%−75% at later time points and remained stable. No statistical differences were observed between D1 and D2 (ns). This pattern suggests an initial fungistatic or fungicidal effect followed by partial regrowth or adaptation. The *C. pungens* exhibited strong and sustained antifungal activity. The effect was dose-dependent only for *A. alternata*, while similar efficacy was observed for *A. solani* and *F. oxysporum*. These results highlight its potential as a natural antifungal agent for plant disease management.

**Figure 14:**
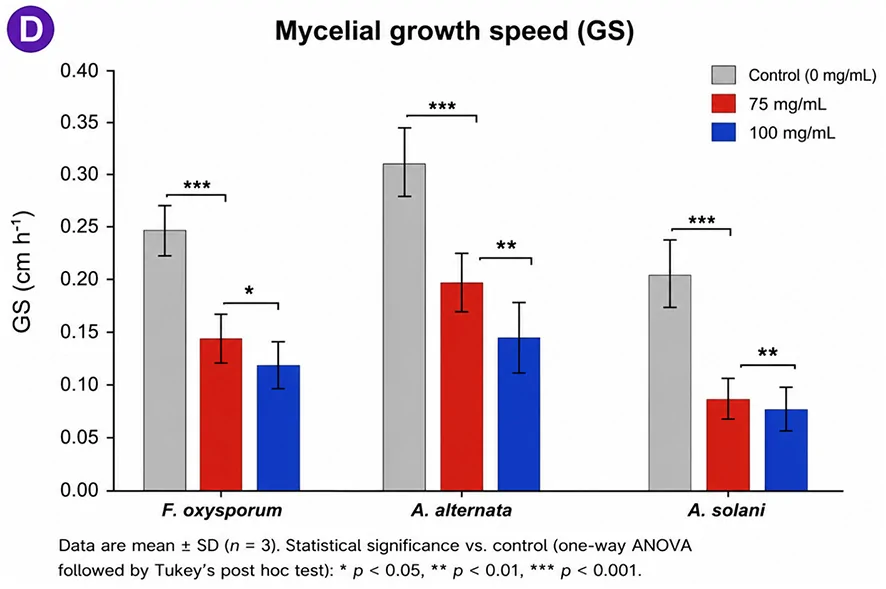
Mycelial growth speed (GS) of different studied fungi against *C. pungens*. Data are mean ± SD (*n* = 3). Statistical significance vs. control (one-way ANOVA followed by Turkey's *post hoc* test). *p < 0.05, **p < 0.05, ***p < 0.001.

Consequently, the antifungal efficacy of *C. pungens* ethanolic extract was further assessed by calculating the mycelial growth speed (GS) of the tested fungi (Figure 14). The growth speed was consistently lower at 100 mg/mL than at 75 mg/mL, confirming a concentration-dependent reduction in fungal growth. Among the tested fungi, *A. solani* exhibited the lowest growth speed (0.079 and 0.091 cm h^−^^1^ at 100 and 75 mg/mL, respectively), indicating the highest susceptibility to the extract. In contrast, *A. alternata* displayed the highest growth rates (0.150 and 0.201 cm h^−1^), suggesting comparatively greater tolerance, whereas *F. oxysporum* showed intermediate values (0.120 and 0.145 cm h^−1^). These findings are consistent with the mycelial inhibition results and further demonstrate that *C. pungens* suppresses fungal growth in a concentration-dependent manner.

The fungicidal activity observed against all three phytopathogenic fungi may be attributed to the combined action of flavonoids and phenolic acids identified by UPLC-ESI-MS/MS. Compounds such as quercetin, luteolin, apigenin, ferulic acid, and myricetin derivatives have been reported to disrupt fungal membrane integrity, interfere with ergosterol biosynthesis, induce oxidative stress, and inhibit key metabolic enzymes (48, 49). Their coexistence in *C. pungens* extract likely contributes to the broad-spectrum antifungal activity observed in this study through synergistic mechanisms.

Antifungal assays showed up to 100% inhibition of *F. oxysporum* at 24 h and sustained inhibition of 70%−78% for other strains. The strong agreement between computational and experimental data confirms the functional relevance of flavonoids. These compounds likely act through enzyme inhibition, radical scavenging, and disruption of microbial membranes. These findings position *C. pungens* as a potent multifunctional agent with applications in pharmaceutical and agricultural systems.

The integration of phytochemical profiling, *in silico* modeling, and experimental validation provides a coherent mechanistic framework for the biological activity of *C. pungens*. UPLC-ESI–MS/MS analysis revealed a polyphenol-rich composition, supported by high total phenolic content (74.91 ± 0.46 mg GAE/g DW) and measurable flavonoid content (5.63 ± 0.08 mg QE/g DW). *In silico* analysis further supported these findings. Myricetin 3-arabinoside exhibited strong binding affinity toward human pancreatic α-amylase, with ΔG_bind reaching up to −65.23 kcal/mol, close to the co-crystal ligand (−78.64 kcal/mol). The compound formed multiple hydrogen bonds with key catalytic residues, including Asp197 and His305, and maintained stable interactions during 300 ns simulations, with ligand RMSD stabilizing around 4.0 Å. These computational results correlate with experimental outcomes. The extract demonstrated strong antioxidant activity, with DPPH IC50 = 0.0706 ± 0.006 mg/mL, H_2_O_2_ scavenging IC50 = 0.1789 ± 0.083 mg/mL, and iron chelation IC50 = 0.1821 ± 0.053 mg/mL. Lipid peroxidation inhibition reached 13.29 ± 0.63% (FTC) and 14.39 ± 6.52% (TBA).

## 4 Conclusion

This study provides a comprehensive evaluation of *C. pungens* as a source of nutritionally relevant bioactive compounds. The extract exhibited a polyphenol-rich phytochemical profile, significant antioxidant activity, and moderate metal-chelating capacity, supporting its antioxidant potential. Molecular docking and molecular dynamics simulations suggested that myricetin 3-arabinoside may contribute to α-amylase inhibition through stable interactions with the enzyme (ΔG_bind = −46.22 kcal/mol). Consistent with these computational findings, the crude extract exhibited concentration-dependent *in vitro* α-amylase inhibitory activity (IC50 = 5.01 mg/mL), indicating the potential of *C. pungens* to modulate carbohydrate digestion and the postprandial glycemic response. From a nutritional perspective, these findings highlight *C. pungens* as a promising source of bioactive phytochemicals for the future development of functional foods and nutraceutical ingredients targeting metabolic health. Although the observed antimicrobial activity may provide additional value for food preservation, the principal nutritional relevance of this species lies in its antioxidant and enzyme-inhibitory properties.

To our knowledge, this study is the first to integrate targeted phytochemical profiling, antioxidant and antimicrobial evaluation, *in vitro* α-amylase inhibition, molecular docking, and molecular dynamics simulations to characterize the nutraceutical potential of *C. pungens*. Collectively, these findings provide a solid foundation for future research. Nevertheless, the present study is limited by the absence of absolute quantitative data for individual bioactive compounds, which constrains the ability to attribute observed biological effects to specific constituents. Quantitative phytochemical analysis, compound isolation and validation, bioavailability studies, toxicity assessment, and *in vivo* efficacy trials are therefore essential next steps before practical nutraceutical or functional food applications can be recommended.

## Data Availability

The raw data supporting the conclusions of this article will be made available by the corresponding author upon request.
